# Prevalence of chronic conditions and multimorbidity among healthcare workers in Zimbabwe: Results from a screening intervention

**DOI:** 10.1371/journal.pgph.0002630

**Published:** 2024-01-23

**Authors:** Claire Jacqueline Calderwood, Edson Marambire, Farirai Peter Nzvere, Leyla Sophie Larsson, Rudo M. S. Chingono, Fungai Kavenga, Nicole Redzo, Tsitsi Bandason, Simbarashe Rusakaniko, Hilda A. Mujuru, Victoria Simms, Palwasha Khan, Celia Louise Gregson, Chiratidzo E. Ndhlovu, Rashida Abbas Ferrand, Katherine Fielding, Katharina Kranzer

**Affiliations:** 1 Faculty of Infectious and Tropical Diseases, Clinical Research Department, London School of Hygiene & Tropical Medicine, London, United Kingdom; 2 The Health Research Unit Zimbabwe, Biomedical Research & Training Institute, Harare, Zimbabwe; 3 Division of Infectious Diseases and Tropical Medicine, Medical Center of the University of Munich, Munich, Germany; 4 Department of Infectious Disease Epidemiology, London School of Hygiene & Tropical Medicine, London, United Kingdom; 5 AIDS & TB Control Programme, Ministry of Health and Child Care, Harare, Zimbabwe; 6 Department of Community Medicine, College of Health Sciences, University of Zimbabwe, Harare, Zimbabwe; 7 Faculty of Medicine and Health Sciences, Child and Adolescent Health Unit, University of Zimbabwe, Harare, Zimbabwe; 8 Data Science Unit, Africa Health Research Institute, Durban, South Africa; 9 Global Health and Ageing Research Unit, Bristol Medical School, University of Bristol, Bristol, United Kingdom; 10 Internal Medicine Unit, College of Health Sciences, University of Zimbabwe, Harare, Zimbabwe; Sree Chitra Tirunal Institute for Medical Sciences and Technology, INDIA

## Abstract

The burden of non-communicable diseases (NCDs) in southern Africa is expanding and is superimposed on high HIV prevalence. Healthcare workers are a scarce resource; yet are vital to health systems. There are very limited studies on the burden of chronic conditions among healthcare workers in Africa, and none exploring multimorbidity (≥2 chronic conditions). We describe the epidemiology of infectious (HIV) and non-communicable chronic conditions, and multimorbidity, among Zimbabwean healthcare workers. Healthcare workers (≥18 years) in eight Zimbabwean provinces were invited to a voluntary, cross-sectional health-check, including HIV, diabetes, hypertension and mental health screening. Statistical analyses described the prevalence and risk factors for multimorbidity (two or more of HIV, diabetes, hypertension or common mental disorder) and each condition. Missing data were handled using multiple imputation. Among 6598 healthcare workers (July 2020–July 2022) participating in the health-check, median age was 37 years (interquartile range 29–44), 79% were women and 10% knew they were living with HIV. Half had at least one chronic condition: 11% were living with HIV, 36% had elevated blood pressure, 12% had elevated HbA1c and 11% had symptoms of common mental disorder. The overall prevalence of multimorbidity was 15% (95% CI: 13–17%); 39% (95% CI: 36–43%) among people aged 50 and older. Whilst most HIV was diagnosed and treated, other chronic conditions were usually undiagnosed or uncontrolled. Limiting our definition of multimorbidity to two or more screened conditions sought to reduce bias due to access to diagnosis, however, may have led to a lower reported prevalence than that found using a wider definition. Half of healthcare workers screened were living with a chronic condition; one in seven had multimorbidity. Other than HIV, most conditions were undiagnosed or untreated. Multisectoral action to implement contextually relevant, chronic disease services in Africa is urgently needed. Specific attention on health workers is required to protect and retain this critical workforce.

## Background

Non-communicable diseases (NCDs) are the leading causes of death globally, with most deaths occurring in low and middle income countries [1]. In particular, diabetes, hypertension, and their consequences, are among the top five causes of death and disability, with their prevalence and impact projected to increase dramatically in the coming decades. In southern Africa, the rapidly increasing impact of NCDs on mortality and years lived with disability is occurring in a context of ongoing high prevalence of HIV, tuberculosis (TB) and nutritional disorders, and a persisting substantial burden of other infections [2]. This presents considerable challenges to fragile health systems.

Shared social and structural risk factors, together with shared biological pathways, mean that chronic conditions coexist within individuals: this phenomenon of multimorbidity, commonly defined as two or more physical or mental health conditions of long duration, magnifies the physical, psychological, social and financial consequences of ill health [3]. The last decade has seen growing awareness of multimorbidity with a suggestion of high prevalence of multimorbidity in Africa (pooled prevalence among adults 28% from a systematic review) [4]. However, population-based data are only available from a few countries and have usually relied on self-reported conditions [4,5], an approach which is likely to underestimate prevalence given that many conditions are undiagnosed [6,7].

The healthcare workforce is the core of any health system: maintaining staff wellbeing is critically important for population health generally, for achieving Sustainable Development Goals, and for our ability to combat future pandemics [8]. Africa faces a healthcare workforce crisis, with a projected shortfall of 6.1 million healthcare workers by 2030 [9]. Alongside a severe HIV epidemic, Zimbabwe has experienced a sustained economic crisis that has severely impacted the health system and resulted in a mass exodus of health workers [10]. The infrastructure for identifying and managing NCDs is weak, resulting in underdiagnosis and undertreatment [11]. As a result of a depleted healthcare workforce, Zimbabwe has been added to the WHO health workforce support and safeguards list [12].

It is likely that chronic diseases and multimorbidity impact on the ability of healthcare workers to continue to work, resulting in illness-related absences, requiring adjustment of work roles, or leading to early retirement. In countries affected by economic migration, staff who remain are generally older and therefore likely to be at higher risk of multimorbidity; increasing the impact of multimorbidity on human resources for health [13]. There are no studies on the prevalence or impact of multimorbidity among healthcare workers in Africa nor of multimorbidity among the general Zimbabwean population. One small study reported on the prevalence of hypertension among healthcare workers in Africa; whilst more reports have considered mental health in the context of the COVID-19 pandemic. Zimbabwean NCD prevalence estimates are either almost 20 years old or restricted to people attending HIV clinics [14].

In the context of the COVID-19 pandemic, we implemented comprehensive health check-ups for healthcare workers in Zimbabwe, to provide access to SARS-CoV-2 testing and to address underlying risk factors for severe COVID-19 (ICAROZ [Impact of the COVID-19 pandemic on healthcare workers and the healthcare system in Zimbabwe]). In this analysis, we aimed to describe the epidemiology of both multimorbidity and the prevalence of individual chronic conditions, among healthcare workers in Zimbabwe.

## Methods

### Study population

From July 2020-July 2022 we invited healthcare workers aged ≥18 years, including domestic and ancillary staff working at 48 hospitals and primary care clinics in Zimbabwe to voluntarily participate in an integrated health-check. Services were provided by a mobile team which remained at each facility until saturation was reached (S1 Text) [10]. In Harare, all hospitals (n = 6) and primary care clinics (n = 20) operating during the intervention period were visited, as well as two private hospitals, three mission hospitals and two non-governmental organization-supported primary care clinics. Between January 2022 and July 2022, as part of the decentralization of COVID-19 care across Zimbabwe, study teams visited health facilities in nine remaining provinces (n = 11 hospitals and n = 4 other facilities) using a similar approach (S1 Text). As a programmatic intervention, no formal sample size calculation was performed, and this analysis used programmatically collected data and therefore was not pre-specified. Table C in S1 Text shows sample sizes required to estimate different prevalences with a certain precision.

### Integrated screening for SARS CoV-2 and chronic conditions

The composition of the screening service has been described elsewhere [10]. Following verbal consent, baseline demographic and health information was collected using a tablet-based questionnaire. Height, weight and blood pressure were measured and screening was offered for SARS-CoV-2, HIV, diabetes, hypertension, visual impairment, common mental disorders, tuberculosis and anaemia. Details of screening procedures are provided in S1 Text. Clients were offered HIV testing if they did not report that they were living with HIV or that they had received a negative test result within the past three months. Testing was either a provider-delivered rapid blood test or an on-site or take-home oral mucosal transudate self-test. Results of take-home tests were not collected. The Shona Symptom Questionnaire (SSQ), a locally validated screening tool for common mental disorders [15], was self-completed as a paper questionnaire or in an audio computer-assisted self-interviewing (ACASI) format (S1 Text). Healthcare workers screening positive for any condition were offered referral to appropriate services. Personal identifiable information (names and phone numbers) was collected for the purposes of referral; this information was collected on paper and stored in locked cabinets accessible to the field team and study coordinators only.

### Definition of chronic conditions

This analysis focused on three non-infectious (mental health, diabetes, and hypertension) and one infectious chronic condition (HIV), using internationally agreed definitions. Elevated blood pressure was defined as systolic BP of at least 140mmHg or diastolic BP of at least 90mmHg; elevated HbA1c was defined as an HbA1c of 6.5% or higher; common mental disorder was defined as an SSQ score of more than eight (Table A in S1 Text). TB was not included as only two people reported previous TB and no one screened positive for TB. We defined ‘screening detected’ as testing positive for a condition when there was no self-reported history and ‘known’ disease as self-report of ever having, or being on treatment for, a condition. ‘Known’ disease was further categorized as ’uncontrolled’ if results of screening were abnormal (e.g. known diabetes and HbA1c ≥6.5%), and ‘controlled’ in the converse (e.g., known diabetes and HbA1c <6.5%) [16]. HIV control was not assessed, therefore we report treatment status only; defined as a self-report of being on antiretroviral therapy (ART).

Multimorbidity was defined as the presence of two or more known or screening-detected chronic conditions in one individual: HIV, elevated HbA1c, elevated blood pressure and/or common mental disorder [3]. Uncontrolled multimorbidity was defined as having two or more conditions, with at least one being uncontrolled or, in the case of HIV, untreated.

### Statistical analysis

A statistical analysis plan was prepared in advance. Analyses were performed in Stata (version 17); descriptive analyses and generation of tables and figures were performed in R (version 4.2.2). Analyses are reported in accordance with Strengthening the Reporting of Observational studies in Epidemiology (STROBE) guidelines (Table W in S1 Text) and recommendations of Sterne et al for epidemiological analysis using multiple imputation.

Missing data for HIV and HbA1c were imputed by chained equations, under a missing at random assumption, with 50 imputed datasets (Table B in S1 Text) [17]. Sensitivity analyses were conducted repeating multiple imputation whilst adjusting with the log odds of undiagnosed HIV among people with unknown test results. This was done because HIV results were likely not missing at random: people who did not test for HIV are potentially at higher HIV risk than those who did [18]. The main scenario considered was one where 14% of the study population living with HIV were not aware of their status; 14% reflects the prevalence of undiagnosed HIV in the adult population of Zimbabwe [19]. The SSQ was initially performed using paper; later individuals were randomised to use a paper or ACASI format. This was implemented after early data suggested a lower-than-expected prevalence of mental health symptoms, for which one hypothesized explanation was underreporting of symptoms on paper-based forms (Table G in S1 Text). The randomised trial showed that SSQ scores were lower among people using paper, compared to ACASI, questionnaires. Multiple imputation was used to adjust scores of people using paper questionnaires to reflect the observed distribution of scores obtained using ACASI (S1 Text).

Primary analyses were conducted on the multiply imputed dataset. The prevalence of multimorbidity and each screened chronic condition (HIV, elevated HbA1c, elevated blood pressure and symptoms of common mental disorders) was calculated within each imputed dataset and combined using Rubin’s rules. Overall, age-band, and sex-stratified prevalence was calculated; and the overlap between different conditions within individuals visualized using Euler diagrams. The yield of screening (proportion of people newly detected at screening) was described, as was the disease control status among people with known disease, stratified by age and sex.

Risk factors for multimorbidity and each chronic condition were described using univariable and multivariable logistic regression. Variables included in multivariable models were defined *a priori* as age, sex, and healthcare occupation category (as a proxy of socio-economic position); a second model additionally adjusted for body mass index (BMI) as a potential mediator of the association between other exposures and elevated HbA1c, blood pressure or multimorbidity. Clustering by health facility was accommodated using robust standard errors.

### Ethics

Ethical approval, including a waiver for written consent was granted by the Medical Research Council of Zimbabwe (MRCZ/A/2627) and the London School of Hygiene & Tropical Medicine (22514). Permission was obtained from medical directorates to operate in health facilities of their jurisdiction. Additional information regarding the ethical, cultural, and scientific considerations specific to inclusivity in global research is included in S2 Text.

## Results

In total, 6598 healthcare workers from 48 facilities participated in the health-check (July 2020–July 2022; Table 1). Most clients participated in Harare (n = 4358). The median number of people enrolled at each hospital or clinic was 148 (interquartile range [IQR] 100–174) and 20 (IQR 6–56) respectively. The median age was 37 (IQR 29–44) years, with 0.5% of people being older than 65 years, the public sector retirement age in Zimbabwe [20]. Most clients (79%) were women and median BMI was 27 kg/m^2^ (IQR 23–31 kg/m^2^). Medical insurance coverage was 65%.

**Table 1:** Characteristics of study population (N = 6598).

	Overall (N = 6598)	Women (N = 5215)	Men (N = 1383)
**Age, years** *	37 (29–44)	37 (29–44)	37 (28–45)
**Employer**			
Public sector	5,713 (87%)	4,564 (88%)	1,149 (83%)
Private sector	338 (5.1%)	242 (4.6%)	96 (6.9%)
NGO/Other	547 (8.3%)	409 (7.8%)	138 (10.0%)
**Occupation**			
Support roles	2,576 (39%)	1,931 (37%)	645 (47%)
Nursing	2,754 (42%)	2,430 (47%)	324 (23%)
Doctors & AHP	593 (9.0%)	407 (7.8%)	186 (13%)
Other	675 (10%)	447 (8.6%)	228 (16%)
**Current cigarette smoker**	175 (2.7%)	19 (0.4%)	156 (11%)
**Medical insurance cover**	4,283 (65%)	3,446 (66%)	837 (61%)
**BMI, kg/m2**			
<18.5, underweight	171 (2.6%)	98 (1.9%)	73 (5.3%)
18.5–24.9, healthy	2,259 (34%)	1,470 (28%)	789 (57%)
25.0–29.9, overweight	2,036 (31%)	1,658 (32%)	378 (27%)
>30.0, obese	2,132 (32%)	1,989 (38%)	143 (10%)
**At least one known medical condition**	1,890 (29%)	1,628 (31%)	262 (19%)
**Known HIV**	675 (10%)	559 (11%)	116 (8.4%)
On ART	670 (99%)	554 (99%)	116 (100%)
**Known anaemia**	6 (<0.1%)	6 (0.1%)	0 (0%)
**Known hypertension**	1,124 (17%)	990 (19%)	134 (9.7%)
**Known diabetes**	239 (3.6%)	213 (4.1%)	26 (1.9%)
**Known mental health condition**	7 (0.1%)	4 (<0.1%)	3 (0.2%)
**Ever had TB**	2 (<0.1%)	1 (<0.1%)	1 (<0.1%)
**Known respiratory condition**	163 (2.5%)	147 (2.8%)	16 (1.2%)
**Known cardiovascular condition**	42 (0.6%)	39 (0.7%)	3 (0.2%)
**Known CKD**	1 (<0.1%)	1 (<0.1%)	0 (0%)
**Ever had cancer**	3 (<0.1%)	2 (<0.1%)	1 (<0.1%)
**Known arthritis**	11 (0.2%)	10 (0.2%)	1 (<0.1%)
**Known epilepsy**	11 (0.2%)	6 (0.1%)	5 (0.4%)
**Known GI condition**	23 (0.3%)	21 (0.4%)	2 (0.1%)

* median and interquartile range. No data were missing for any of the variables presented. Occupation was categorized into nurses (including midwives), doctors and allied health professionals (i.e. other clinical roles requiring a higher degree), clinical support roles (for example community health workers, housekeeping, or nursing assistants) and other (including administrators and security).

Abbreviations

AHP = allied health professional; ART = anti-retroviral therapy; BMI = body mass index (kilograms / meter squared); CKD = chronic kidney disease; HIV = human immunodeficiency virus; GI = gastrointestinal; N = number; NGO = non-governmental organisation; TB = tuberculosis

All included individuals were screened for blood pressure and common mental disorder (Table D in S1 Text) and 84% (n = 5532) had HbA1c measured. Acceptance of HbA1c screening was high, but test stock outs resulted in 16% (n = 1066) with unknown HbA1c (Fig A in S1 Text). HIV status was known (i.e., self-reported ever testing positive or a self-reported negative test in the past three months) for 62% (n = 4063) of individuals; 14% of those with unknown HIV status (n = 922) opted for offsite HIV testing and 24% (n = 1613) declined HIV testing. Fifty-one percent of clients (n = 3390) had complete data for both HIV and HbA1c. The distribution of missing data for HIV status and HbA1c are shown in Tables E and F in S1 Text; and distribution of observed and imputed values are shown in Tables H and I in S1 Text. All subsequent analyses were conducted using the imputed dataset.

### Prevalence of chronic conditions and multimorbidity

The prevalence of multimorbidity was 15% (95% confidence interval [CI] 13–17%; Table 2 and Tables J–L in S1 Text). Eleven percent of healthcare workers had previously diagnosed or screening detected HIV, 36% elevated blood pressure, 12% elevated HbA1c, and 10% symptoms of common mental disorders. The prevalence of at least one of the four chronic conditions among the study population was 52% (95%CI 50–55%), with 34% (95%CI 31–37%) identified with least one previously undiagnosed chronic condition by screening. Among people with multimorbidity, 88% had hypertension. The most common disease combinations were hypertension and diabetes (46%), hypertension and HIV (30%) and hypertension and mental health (27%; Table S in S1 Text).

**Table 2:** Prevalence of known and screening-detected chronic conditions and multimorbidity.

	Complete records (N = 3390)*	Multiple imputation (N = 6598)*		
	Total % (95% CI)	Total % (95% CI)	Known % (95% CI)	Screening detected % (95% CI)
**OVERALL**				
Multimorbidity	18.1 (15.0–21.6)	14.9 (12.7–17.1)	–	–
HIV	17.2 (13.6–21.4)	11.2 (8.6–13.7)	10.2 (7.7–12.7)	0.9 (0.4–1.4)
Elevated BP	37.7 (34.9–40.5)	36.0 (33.5–38.5)	17.0 (14.9–19.1)	18.9 (15.5–22.3)
Elevated HbA1c	12.1 (10.0–14.4)	11.9 (10.1–13.7)	3.6 (2.8–4.4)	8.3 (6.8–9.8)
Common mental disorder	10.5 (8.0–13.5)	10.7 (8.9–12.5)	0.1 (0.1–0.5)	10.7 (8.9–12.5)
**WOMEN**				
Multimorbidity	18.7 (15.4–22.4)	15.4 (13.2–17.7)	–	–
HIV	17.9 (14.2–22.3)	11.7 (9.1–14.3)	10.7 (8.2–13.3)	1.0 (0.4–1.5)
Elevated BP	37.2 (34.7–39.7)	36.1 (34.1–38.2)	19.0 (16.5–21.5)	17.1 (14.0–20.2)
Elevated HbA1c	12.5 (10.4–15.1)	12.3 (10.4–14.2)	4.1 (3.2–4.9)	8.2 (6.7–9.7)
Common mental disorder	10.7 (8.2–13.8)	11.0 (9.1–12.9)	0.1 (0.0–0.2)	11.0 (9.1–12.9)
**MEN**				
Multimorbidity	15.8 (11.7–20.9)	12.9 (9.7–16.0)	–	–
HIV	14.5 (10.5–19.6)	9.2 (6.2–12.2)	8.4 (5.5–11.3)	0.8 (0.0–1.7)
Elevated BP	39.5 (33.9–45.4)	35.4 (30.1–40.8)	9.7 (7.8–11.6)	25.7 (20.3–31.2)
Elevated HbA1c	10.3 (7.5–14.0)	10.4 (7.8–13.0)	1.9 (0.8–2.9)	8.5 (6.2–10.8)
Common mental disorder	9.7 (7.0–13.3)	9.5 (7.1–12.0)	0.2 (0.0–0.5)	9.5 (7.1–12.0)

Clients were considered to have a condition if they either self-reported or screened positive during the study. Screening positive was defined as follows. Elevated blood pressure (BP): Systolic BP ≥140mmHg or diastolic BP ≥90mmgHg. Elevated HbA1c: ≥6.5%. Common mental disorder: Shona Symptom Questionnaire Score≥8.

* Presented as % prevalence (95% confidence interval). Estimates are adjusted for health facility-level clustering using robust standard errors.

**Abbreviations:** BP = blood pressure; HbA1c = haemoglobin A1C; HIV = human immunodeficiency virus; GI = gastrointestinal; N = number; NGO = non-governmental organisation; TB = tuberculosis.

**Fig 1:**
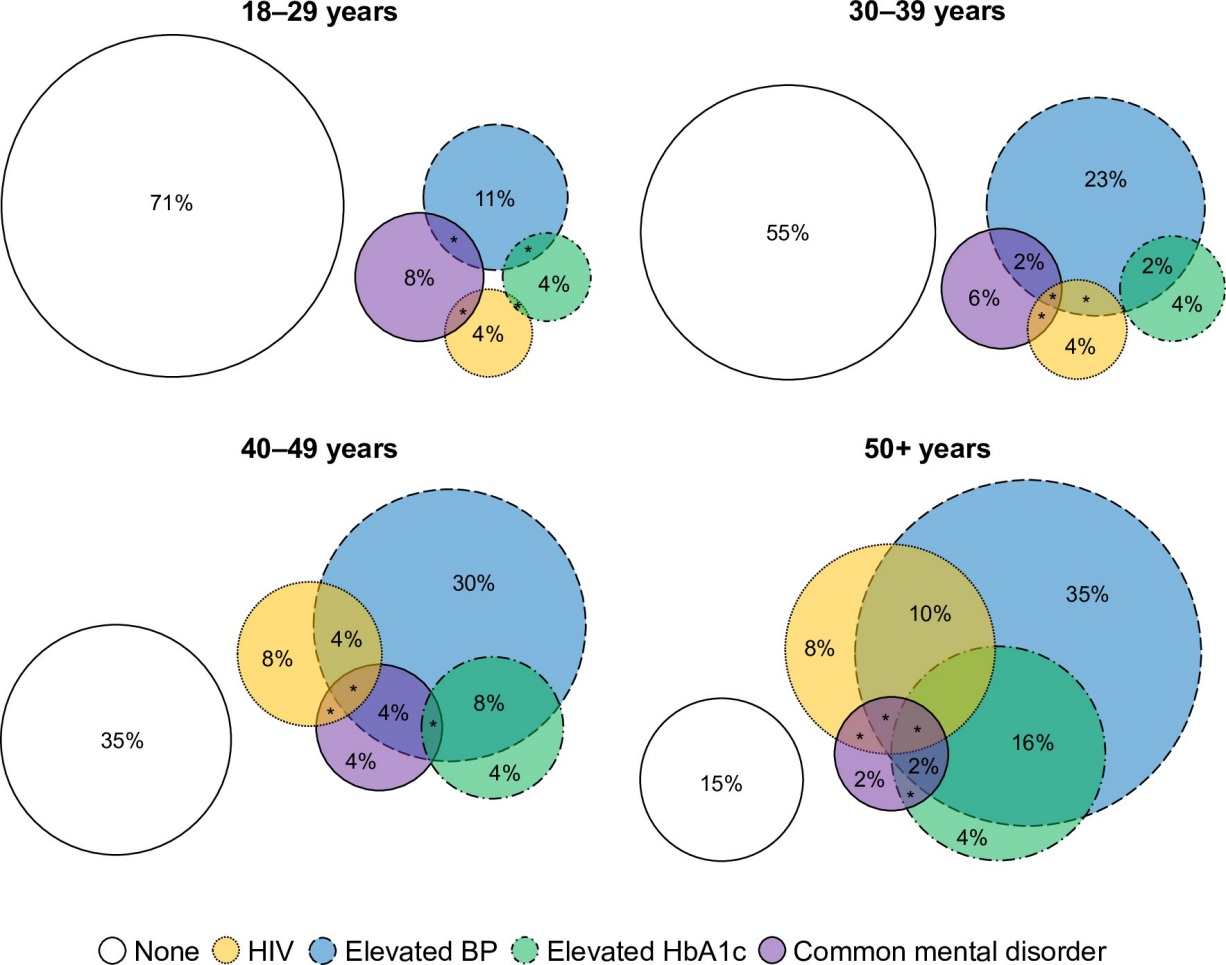
Euler diagrams illustrating prevalence and clustering of chronic conditions by age categories, in multiply imputed dataset (N = 6598). * indicates prevalence ≤1%. Conditions are indicated by colour and line type. Non-intersecting white circles = no conditions; yellow circles with dotted lines = HIV; blue circles with dashed lines = elevated blood pressure (BP); green circles with dot/dash lines = elevated HbA1c; purple circles with solid lines = common mental disorder.

The prevalence of multimorbidity was 15% (95%CI 13–18%) among women and 13% (95%CI 10–16%) among men, and increased with age (Fig 1 and Table J in S1 Text). Amongst those aged ≥50 years there was considerable overlap of all chronic conditions, with 39% of this age group having multimorbidity (95%CI 36–43%) and 7% having three or four conditions (‘complex multimorbidity’ [21]).

Prevalence of multimorbidity among people who were overweight (14%) or obese (22%) was higher than among people with normal BMI (9%). Among people with normal BMI, 24% had hypertension and 7% had diabetes; prevalences of both hypertension and diabetes were higher among people who were overweight or obese (Table K in S1 Text).

Sensitivity analyses suggested that in a scenario where 14% of people with HIV did not know their status, prevalence estimates for HIV would have been 0.6% higher, and multimorbidity estimates would have been the same (Table V in S1 Text).

### New diagnoses and management of chronic conditions

The prevalence of screening detected elevated HbA1c and elevated blood pressure was 8.2% and 19% respectively (i.e., 69% and 53% of all disease, respectively, was newly detected at screening), whilst prevalence of screening detected symptoms of common mental disorders was 10.7% (i.e., over 99% of people with a positive screening result for common mental disorders did not report having a mental health disorder). This contrasted with HIV where the prevalence of newly detected HIV was 0.9% (i.e., 8% of all HIV was not previously known).

**Fig 2:**
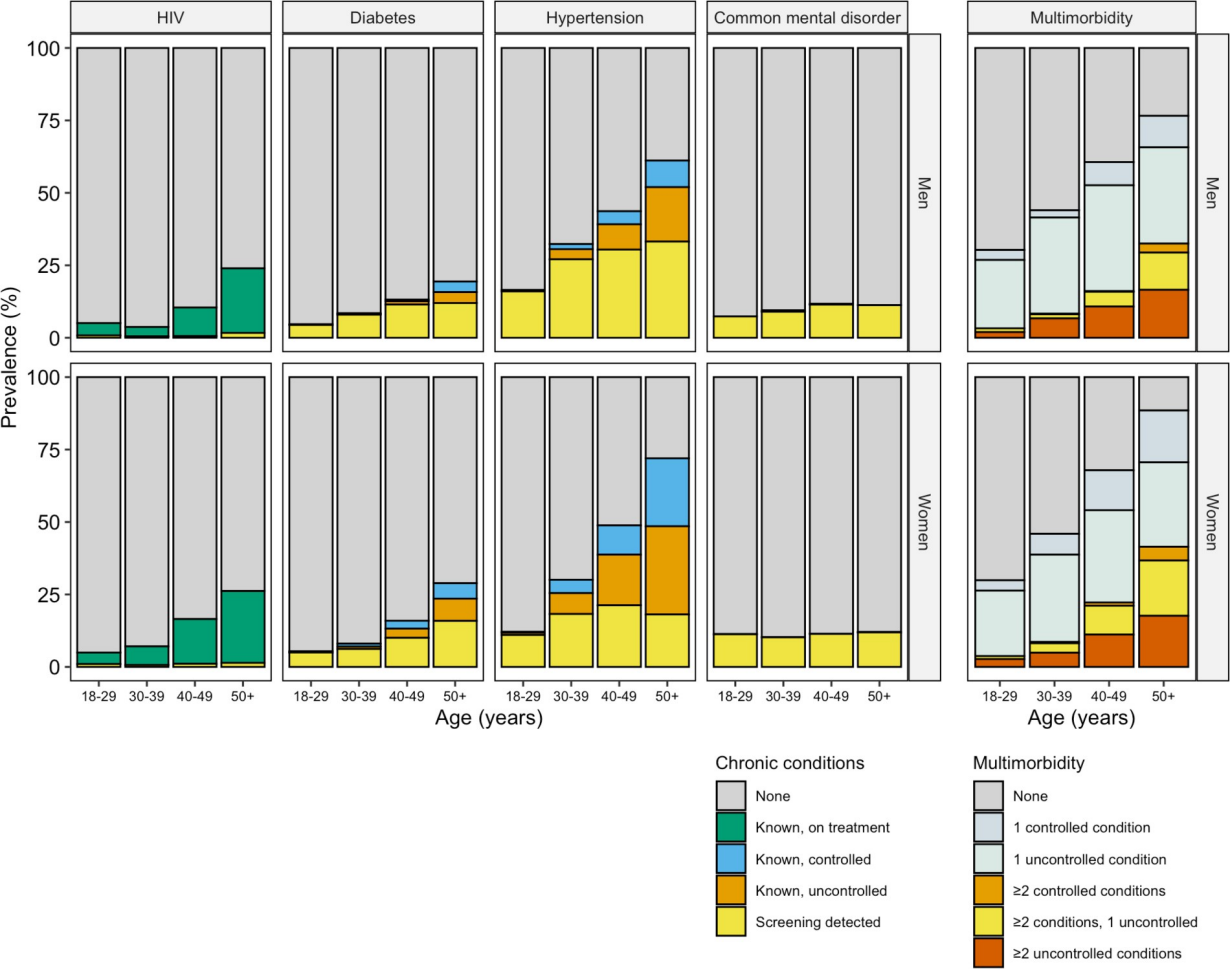
Disease control status of chronic conditions and multimorbidity, in multiply imputed dataset (N = 5215 women / 1383 men). Clients were considered to have a condition if they either self-reported or screened positive during the study. Screening positive was defined as follows. Hypertension: Systolic BP ≥140mmHg or diastolic BP ≥90mmgHg. Diabetes: ≥6.5%. Common mental disorder: Shona Symptom Questionnaire Score ≥8. In the plot of multimorbidity (right panel), included conditions are HIV, diabetes, hypertension, and common mental disorders. Multimorbidity is defined as two or more conditions coexisting in an individual (orange, yellow or red). Disease control is defined as having a previous history of the condition, but a normal result during screening (e.g., known diabetes but HbA1c <6.5% on testing). HIV control was not assessed, HIV on treatment is included as a ‘controlled’ condition in the multimorbidity figure.

Among people with known disease, 94% of people with diabetes, 92% of people with hypertension and over 99% of people with HIV reported being on treatment. Tests showed that 47% of people with known diabetes and 39% of people with known hypertension had controlled disease (Fig 2 and Tables O and P in S1 Text).

### Risk factors for chronic conditions and multimorbidity

After adjustment for age and occupation, women were more likely to have multimorbidity and each of elevated HbA1c, HIV and symptoms of common mental disorders compared to men. No association between sex and overall prevalence of elevated blood pressure was observed (Table 3). Across both univariable and multivariable models, increasing age was associated with increased odds of all conditions other than common mental disorders. After additional adjustment for BMI, as a proposed mediator of the association between the exposures of age or sex and outcomes of multimorbidity, elevated HbA1c, and elevated blood pressure, the strong associations between age and each outcome persisted. After adjustment for BMI, there was no evidence for increased risk of multimorbidity or elevated HbA1c among women compared to men; whilst men were at a higher risk of elevated blood pressure than women (OR 1.23 [95%CI 1.06–1.43]; Table S in S1 Text).

**Table 3:** Univariable and multivariable risk factors for multimorbidity and its component conditions, after multiple imputation (N = 6598).

	Level	n/N*	Unadjusted OR (95%CI)	p†	Adjusted OR (95%CI)	p†
**MULTIMORBIDITY**						
Sex	Women	500/2679	–	0.02	–	0.007
	Men	112/711	0.81 (0.64–1.02)		0.72 (0.57–0.92)	
Age, years	18–29	29/765	–	<0.001†	–	<0.001†
	30–39	114/1155	2.50 (1.83–3.43)		2.40 (1.74–3.31)	
	40–49	220/890	7.07 (5.16–9.70)		6.59 (4.80–9.05)	
	50+	249/580	17.4 (12.8–23.6)		16.3 (12.0–22.1)	
Occupation	Support roles	336/1383	–	<0.001	–	<0.001
	Nursing	179/1360	0.53 (0.43–0.67)		0.71 (0.56–0.91)	
	Doctors & AHP	39/306	0.45 (0.33–0.62)		0.69 (0.52–0.92)	
	Other	58/341	0.72 (0.54–0.96)		0.69 (0.56–0.86)	
BMI category	Underweight	12/86	1.01 (0.61–1.67)	<0.001†		
	Healthy	139/1114	–			
	Overweight	182/1062	1.62 (1.27–2.05)			
	Obese	279/1128	2.71 (2.07–3.54)			
**HIV**						
Sex	Women	479/2679	–	0.03		<0.001
	Men	103/711	0.76 (0.60–0.97)		0.62 (0.49–0.79)	
Age, years	18–29	63/765	–	<0.001†		<0.001†
	30–39	117/1155	1.33 (0.84–2.12)		1.14 (0.73–1.77)	
	40–49	204/890	3.48 (2.24–5.41)		2.56 (1.68–3.91)	
	50+	198/580	6.60 (4.21–10.4)		4.97 (3.19–7.74)	
Occupation	Support roles	416/1383	–	<0.001	–	<0.001
	Nursing	110/1360	0.23 (0.16–0.31)		0.26 (0.19–0.38)	
	Doctors & AHP	21/306	0.17 (0.11–0.25)		0.22 (0.15–0.33)	
	Other	35/341	0.31 (0.19–0.50)		0.31 (0.20–0.49)	
BMI category	Underweight	18/86	1.00 (0.63–1.60)	0.8		
	Healthy	218/1114	–			
	Overweight	182/1062	0.95 (0.76–1.18)			
	Obese	164/1128	0.89 (0.69–1.14)			
**ELEVATED BLOOD PRESSURE**						
Sex	Women	996/2679	–	0.8	–	0.6
	Men	281/711	0.97 (0.81–1.17)		0.96 (0.80–1.15)	
Age, years	18–29	106/765	–	<0.001†	–	<0.001†
	30–39	362/1155	2.90 (2.49–3.37)		2.95 (2.53–3.46)	
	40–49	416/890	6.03 (5.10–7.14)		6.40 (5.43–7.53)	
	50+	393/580	15.02 (11.58–19.47)		16.04 (12.54–20.51)	
Occupation	Support roles	530/1383	–	<0.001	–	<0.001
	Nursing	500/1360	0.84 (0.73–0.97)		1.26 (1.12–1.41)	
	Doctors & AHP	105/306	0.75 (0.59–0.96)		1.29 (0.99–1.67)	
	Other	142/341	1.19 (1.01–1.40)		1.29 (1.09–1.54)	
BMI category	Underweight	20/86	0.76 (0.58–1.00)	<0.001†		
	Healthy	283/1114	–			
	Overweight	392/1062	1.75 (1.44–2.12)			
	Obese	582/1128	3.26 (2.62–4.06)			
**ELEVATED HBA1C**						
Sex	Women	336/2679	–	0.1	–	0.07
	Men	73/711	0.83 (0.66–1.04)		0.79 (0.62–1.02)	
Age, years	18–29	36/765	–	<0.001†	–	<0.001†
	30–39	91/1155	1.60 (1.19–2.14)		1.58 (1.18–2.11)	
	40–49	127/890	3.28 (2.41–4.46)		3.30 (2.42–4.50)	
	50+	155/580	6.59 (4.75–9.13)		6.67 (4.83–9.22)	
Occupation	Support roles	178/1383	–	<0.001	–	0.7
	Nursing	152/1360	0.83 (0.69–0.99)		1.08 (0.91–1.29)	
	Doctors & AHP	31/306	0.67 (0.51–0.88)		0.97 (0.72–1.32)	
	Other	48/341	1.07 (0.83–1.39)		1.13 (0.86–1.49)	
BMI category	Underweight	3/86	0.82 (0.37–1.82)	<0.001†		
	Healthy	75/1114	–			
	Overweight	130/1062	1.78 (1.43–2.22)			
	Obese	201/1128	2.97 (2.31–3.80)			
**COMMON MENTAL DISORDER**						
Sex	Women	286/2679	–	0.2	–	0.08
	Men	69/711	0.85 (0.66–1.09)		0.80 (0.62–1.04)	
Age, years	18–29	61/765	–	0.3	–	0.6
	30–39	108/1155	0.96 (0.73–1.25)		0.91 (0.69–1.21)	
	40–49	119/890	1.11 (0.82–1.50)		1.01 (0.74–1.39)	
	50+	67/580	1.15 (0.79–1.67)		1.04 (0.72–1.52)	
Occupation	Support roles	204/1383	–	0.2	–	0.2
	Nursing	105/1360	0.70 (0.52–0.93)		0.69 (0.51–0.92)	
	Doctors & AHP	22/306	0.72 (0.48–1.08)		0.73 (0.48–1.11)	
	Other	24/341	0.75 (0.51–1.12)		0.77 (0.52–1.14)	
BMI category	Underweight	11/86	1.08 (0.60–1.92)	0.9		
	Healthy	112/1114	–			
	Overweight	107/1062	1.00 (0.79–1.27)			
	Obese	125/1128	1.00 (0.79–1.27)			

Clients were considered to have a condition if they either self-reported or screened positive during the study. Screening positive was defined as follows. Elevated blood pressure (BP): Systolic BP ≥140mmHg or diastolic BP ≥90mmgHg. Elevated HbA1c: ≥6.5%. Common mental disorder: Shona Symptom Questionnaire Score >8. Body mass index (BMI) was defined according to World Health Organization (WHO) definitions: Underweight: <18.5 kg/m^2^; Normal range: 18.5–25 kg/m^2^; Overweight: 25–30 kg/m^2^; Obese: 30+ kg/m^2^.

All estimates are adjusted for health facility-level clustering using robust standard errors. aOR is additionally adjusted for variables shown in the table (sex, age, occupation and BMI category).

* Complete case numbers (N = 3390). P value for association presented

† symbol indicates p value for linear trend <0.001.

**Abbreviations**: n/N = number with the outcome / total number in stratum indicated; OR = odds ratio; aOR = adjusted OR.

Higher BMI category was associated with increased odds of multimorbidity and of elevated HbA1c or elevated blood pressure but was not associated with HIV or common mental disorder, in univariable models. After adjustment for age, sex and occupation, the associations between BMI and multimorbidity, elevated HbA1c and elevated blood pressure persisted (Table S in S1 Text).

People living with HIV were less likely to have elevated blood pressure or elevated HbA1c compared to people without HIV, after adjustment for age, sex, and occupation (Table T in S1 Text). Among all people with elevated HbA1c or blood pressure, respectively, the odds of having been diagnosed with diabetes/hypertension prior to accessing the health-check, were similar among people living with HIV and in care, compared to people who were not living with HIV (Table U in S1 Text).

## Discussion

This study is the first systematic evaluation of multimorbidity and key chronic conditions among healthcare workers in Africa. Importantly, we considered mental health as a long-term condition and component of multimorbidity. We demonstrated a high prevalence of elevated HbA1c, elevated blood pressure, HIV, and common mental disorders. Over half the healthcare workers were living with at least one chronic condition, whilst considerable overlap between conditions, particularly among those aged 50 and older, resulted in a significant burden of multimorbidity. However, the majority of healthcare workers were under the age of 45, with potentially more than 20 years left of their working lives [20]. Amongst this group, overweight and obesity were very common, particularly among women, as were individual chronic conditions. Complications or additional morbidities are likely to develop during the working lives of these younger healthcare workers without effective treatment and prevention. Whilst 93% of healthcare workers living with HIV knew their status and 99% of those were on treatment, fewer than half of those with elevated HbA1c or blood pressure were aware of their condition (31% and 47% respectively). People who were in regular care for HIV were no more likely to know about their elevated HbA1c or blood pressure, compared to those who were not, suggesting a lack of integration of NCD care into HIV clinics. Overall, half of people with known diabetes and two thirds of people with known hypertension had uncontrolled disease. Almost all common mental disorders were previously unrecognized. This is despite almost two thirds of all healthcare workers having access to medical insurance.

Morbidity and premature mortality are recognised as contributing to the crisis in human resources for health in Africa;[9] however, occupational health services traditionally focus on specific transmissible conditions (e.g. HIV, TB, viral hepatitis) rather than delivering holistic health care. This study has provided the first evidence on the threat of multimorbidity to human resources for health in Africa. The very high burden of undiagnosed or uncontrolled chronic conditions identified here illustrates an unmet need for chronic disease services and mental health support [22]. It is likely that chronic conditions are impacting on healthcare workers’ wellbeing and ability to work effectively. Preventative, diagnostic and treatment services for chronic conditions may enable healthcare staff to continue to work and may aid retention: in a discrete choice experiment, South African healthcare workers preferred medical aid packages over a similar salary increase [23]. Healthcare workers have good health literacy and, theoretically, access to healthcare; hence, it is likely that the unmet need for chronic disease services among the general Zimbabwean population is even higher than that reported here.

Improving access to diagnostic services or screening programmes such as ours are, however, insufficient to prevent morbidity and mortality. It will also be necessary to address structural and social determinants of health and to support people’s ability to change their lifestyle, access treatment, remain in care and adhere to continuous treatment [6,7,11]. Our findings support calls for integrated strategies to co-address diabetes, hypertension, and overweight/obesity in Africa. Such approaches will need to move beyond the biomedical to include effective communication and multi-sectoral approaches that address commercial and environmental barriers to a ‘healthy lifestyle’ [24].

The impressive implementation, scale-up and decentralization of HIV care in Africa, supported by adequate funding and innovative care models, has demonstrated that delivering long-term, decentralized care at scale in Africa is feasible, and could provide a template for other chronic conditions. Unfortunately, NCD services are not established to the same extent. In Zimbabwe, HIV care and treatment is freely available at all government primary care clinics and medication stock outs are rare. In contrast, people with NCDs pay for clinic fees and medications, whilst lack of NCD care at primary care level results in considerable transport costs [11]. These are well recognised barriers to effective NCD management [24]. Innovative approaches, including service integration and multimorbidity-sensitive approaches, supported by increased funding and awareness among healthcare workers and communities, are needed to tackle the converging epidemics of NCDs and HIV. To adequately care for people with multimorbidity, programmes will need to move away from siloed, disease-specific models, towards integrated, person-centred care. Addressing risk factors such as overweight/obesity or high salt and sugar intake, together with optimal management and secondary prevention among younger people with chronic conditions may prevent the development of multimorbidity.

The marked heterogeneity in multimorbidity estimates and frequent reliance on self-reported conditions makes it challenging to draw comparisons between studies. Our estimate of multimorbidity prevalence fell within the range described in LMIC by systematic reviews (13–87%) [4,5], was slightly higher than a recent study (including HIV, TB, hypertension and diabetes) in South Africa, and considerably higher than estimates from Malawi (including overweight/obesity, diabetes and hypertension) [6,16]. The prevalence of elevated HbA1c described among healthcare workers was more than double the estimated diabetes prevalence among African adults (12% vs 4.5%) but similar to that described in the Zimbabwe STEPS survey in 2005 [14,25]. The prevalence of hypertension was similar to regional estimates but higher than that reported in the STEPS survey.

To our knowledge no other study has described multimorbidity among African healthcare workers and very few have screened for conditions other than transmissible diseases (such as HIV and TB), or for mental health in the context of the COVID-19 pandemic. One small study reported high prevalence (but low awareness) of hypertension among healthcare workers in Sierra Leone, with prevalence similar to the general population [26]. Described risk factors for multimorbidity were similar to reports from other populations [4]. Prevalence of multimorbidity, HIV, diabetes and common mental disorder were higher among women compared to men [16], whilst men were more likely to have undiagnosed disease than women. The higher odds of multimorbidity among women was no longer observed after adjusting for BMI, suggesting that this association may be mediated by higher prevalence of overweight and obesity among women.

Strengths of our study include the large sample size and not restricting the study population to individuals seeking care, on which most studies of multimorbidity have relied. Screening enabled estimation of both diagnosed and undiagnosed disease and included common mental disorders. Mental health is infrequently considered in multimorbidity studies [4] despite being associated with many physical health conditions and being fundamental for personal wellbeing. Using 2018 estimates of the Zimbabwean healthcare workforce our service reached 13% of healthcare workers (n = 6652/50414 [9]); however, over the intervening period the ‘exodus’ of Zimbabwean healthcare workers has accelerated and therefore current numbers of healthcare workers are likely to be lower.

Limitations of this study include a multimorbidity definition based on only four conditions (HIV, elevated blood pressure, elevated HbA1c and common mental disorders). This does not reflect the broad spectrum of potential conditions contributing to multimorbidity. Whilst visual impairment and anaemia screening was offered as part of the health check, this was implemented part way through the study and therefore there were considerable missing data. These variables were therefore not included in the analysis. ‘Counting’ diseases does not recognise that multimorbidity is a person- rather than disease-centred process [27]. Our choice of conditions was informed by practical and ethical considerations (such as the need for treatment services to be available); they are among the most prevalent components of multimorbidity with hypertension and diabetes having been described as ‘starting conditions’ for multimorbidity [28]. Studies using ‘wider’ definitions of multimorbidity report higher prevalence than described here [4,29].

Further limitations include a healthy worker effect [30] and voluntary participation potentially resulting in selection bias of those considering themselves at increased risk. This could have led to over- and underestimation of disease prevalence. Unfortunately, data on the total number of staff working at each health facility were not available. However, screening was free, various strategies (including advertisements, meetings, daily out-reach to clinical areas and Saturday shifts) were employed to ensure all staff knew about the service and could access it. Process evaluation suggested high acceptability and we think it is likely that most healthcare workers at participating facilities took part in the service. Our study population was mostly women, in keeping with the composition of the health workforce [31]. The pragmatic, cross-sectional design of the health-checks means that our estimates were based on testing at a single time point using point-of-care tools and there were considerable missing data for HIV and HbA1c. Previous studies have suggested that blood pressure measurement on a single day may overestimate hypertension prevalence [32]. HbA1c has high specificity but low sensitivity (58%) for diabetes in Africa; HbA1c-based screening may therefore underestimate diabetes prevalence [33]. Whilst we suggest HbA1c results are likely to be missing at random, this is not the case for HIV. The recent ZimPHIA survey suggested 13% adults living with HIV in Zimbabwe do not know their status; reassuringly, simulation of this scenario in sensitivity analyses made no difference to our estimates of multimorbidity prevalence [19].

In conclusion, this study demonstrates an alarmingly high prevalence of chronic conditions among Zimbabwean healthcare workers. The majority of NCDs are undiagnosed or uncontrolled and multimorbidity is the norm amongst people aged 50 and older. These data support urgent calls for multisectoral action and investment to develop contextually relevant, chronic disease services in Africa, and highlight the need for specific attention on health workers. Unaddressed, multimorbidity is likely to present an enormous challenge to fragile African health systems in the coming decades.

## Supporting information

S1 Text Supplementary methods, results and completed STROBE checklist. (DOCX)

S2 Text Inclusivity questionnaire. (DOCX)
