# Supplementary material for: Prevalence of chronic conditions and multimorbidity among healthcare workers in Zimbabwe: Results from a screening intervention
Source: PLOS Glob Public Health. 2024 Jan 23;4(1):e0002630. doi: 10.1371/journal.pgph.0002630 (PMC10805297; doi:10.1371/journal.pgph.0002630)
Supplement: S1 Text — (DOCX) [file pgph.0002630.s001.docx]

SUPPLEMENTARY MATERIALS

# Prevalence of chronic conditions and multimorbidity among healthcare workers in Zimbabwe: results from a screening intervention

**Authors:**

Claire Jacqueline Calderwood^1,2^*, Edson Marambire^2,3^, Farirai Peter. Nzvere^2,5^, Leyla Sophie Larsson^2,3^, Rudo MS. Chingono^2^, Fungai Kavenga^2,4^, Nicole Redzo^2^, Tsitsi Bandason^2^, Simbarashe Rusakaniko^6^, Hilda A. Mujuru^7^, Victoria Simms^1,8^, Palwasha Khan^1,8^, Celia L Gregson^2,9^, Chiratidzo E. Ndhlovu^10^, Rashida Abbas Ferrand^1,2^, Katherine Fielding^5^, Katharina Kranzer^1,2,3^

1. Faculty of Infectious and Tropical Diseases, London School of Hygiene & Tropical Medicine, United Kingdom
2. The Health Research Unit Zimbabwe, Biomedical Research & Training Institute, Zimbabwe
3. Division of Infectious Diseases and Tropical Medicine, Medical Center of the University of Munich, Munich, Germany
4. AIDS & TB Control Programme, Ministry of Health and Child Care, Harare, Zimbabwe.
5. Department of Infectious Disease Epidemiology, London School of Hygiene & Tropical Medicine, United Kingdom
6. Department of Community Medicine, College of Health Sciences, University of Zimbabwe, Harare, Zimbabwe
7. Child and Adolescent Health Unit, University of Zimbabwe Faculty of Medicine and Health Sciences, Harare, Zimbabwe
8. Data Science Unit, Africa Health Research Institute, Durban, South Africa
9. Global Health and Ageing Research Unit, Bristol Medical School, University of Bristol, United Kingdom
10. Internal Medicine Unit, University of Zimbabwe College of Health Sciences, Harare, Zimbabwe

***** claire.calderwood2@lshtm.ac.uk

# SUPPLEMENTARY METHODS

## Implementation of screening

For facilities in Harare, the screening service was set up at each site, following preliminary meetings with members of the management team. Meetings, posters and daily in-reach to all clinical areas aimed to maximise uptake of the intervention. Saturation was defined as having been achieved when fewer than five people attended the service in a day, and in-reach activities suggested there were no further staff members who were interested in participating.

For facilities visited as part of COVID-19 care decentralisation activities, all four hospitals in Bulawayo (the second largest city in Zimbabwe) and seven provincial hospitals were visited. There are a total of 10 provinces in Zimbabwe, including Harare and Bulawayo. Mashonaland Central province is adjacent to Harare. The provincial hospital was not visited due to timing constraints, however four other health facilities in that province were visited instead. Overall, all 10 provinces of Zimbabwe were reached by screening activities. For sites outside Harare, the team were based at a facility for a total of 10 consecutive days (including one Saturday/Sunday). This duration of intervention was informed by estimates of the size of facilities and experience at previous sites.

No formal estimates of the number of staff working at each facility was available, therefore the coverage of the intervention is not known. Process evaluation suggested that screening was very popular and uptake was high; including among healthcare workers who had privately purchased health insurance. Healthcare workers perceived that services covered by medical insurance were limited and required co-payments for shortfalls, few participants (including those with health insurance) reported similar screening services being available elsewhere.

## Screening procedures

Screening used the following measurements:

- Diabetes: point-of-care HbA1c, A1c Care (SD Biosensor, Republic of Korea)
- HIV: either a provider-delivered rapid blood test (Alere Determine HIV1/2, Alere, United States of America [USA] & Chembio Diagnostics, USA), according to national guidelines, or oral mucosal transudate self-test (Oraquick, Orasure Technologies, USA), which healthcare workers could take on-site or at home.^1^
- Hypertension (three automated blood pressure readings taken at five minute intervals, Omron M1 [Omron, Japan])
- Common mental disorder: Shona Symptom Questionnaire (SSQ).^2^ The SSQ was delivered as a self-completed paper questionnaire or audio computer-assisted self-interviewing (ACASI) format (Supplementary methods).
- SARS-CoV-2: symptom screened followed by polymerase chain reaction [PCR] or lateral flow assay)
- Tuberculosis: World Health Organization [WHO] symptom screening followed by Xpert MTB/Rif Ultra [Cepheid, USA] if positive^3^.
- Visual impairment (distance vision assessment Peek Acuity [Peek Vision, United Kingdom])

## Administration of mental health screening

The Shona Symptom Questionnaire (SSQ) is a locally validated mental health screening tool used widely in Zimbabwe.^4^ It is comprised of 14 questions screening for ‘common mental disorder’ including anxiety and depression (however these two are not differentiated by the tool). A score of eight or more is considered a positive result. In the first iteration of the serbice, the SSQ was completed on paper by clients in a private area whilst waiting to have their anthroprometric measurements and done and see the nurse. Early data found a 9% prevalence of common mental disorder which the study team found surprising in the context of the very challenging working conditions faced by the clients. This was further explored by switching to an audio computer-assisted self-interviewing (ACASI) format and including other mental health measures. These findings will be reported in detail elsewhere (Simms V, unpublished), however they suggest that use of paper-based questionnaires resulted in lower prevalence of common mental disorder compared to ACASI.

## Definitions

Chronic condition definitions can be found in Table 1.1. Whilst other conditions (e.g. visual impairment, anaemia, SARS-CoV-2) were also included as part of the screening intervention we limited our analysis to HIV, elevated BP, elevated HbA1c and common mental disorders. Several considerations were taken into account when developing this analysis:

- This was a programmatic intervention which was iteratively refined based on process evaluation from clients and service providers. It was this feedback that led to implementation of visual impairment and anaemia screening, part way through the study. As a result, there was significant missing data (30% for visual impairment and 59% for anaemia). Given the volume of missing data for HIV and HbA1c we did not consider it appropriate to additionally impute missing data in visual impairment and haemoglobin, which would have been required for their inclusion.
- Diabetes and hypertension (sometimes also with obesity) are the most commonly described NCDs in Africa, whilst few robust estimates of other NCDs exist.^5,6^ We therefore prioritized these conditions as part of our definition. They were also the most prevalent NCDs in our dataset (prevalence of visual impairment 6.9% and anaemia 5.2%; complete case records).
- Whilst anaemia screening was included, we did not have any data on the cause of anaemia, making this difficult to interpet.

Exposures of interest included age, sex, occupation, smoking (current smoker vs not), whether someone had medical aid insurance and body mass index (BMI). For analysis, age and BMI were categorised to aid clinical interpretability of results. Age was categorized into 4 approximate 10 year age bands (18–29 years, 30–39 years, 40–49 years and 50 years and older. BMI was defined according to World Health Organization (WHO) definitions (Underweight: <18.5 kg/m^2^; Normal range: 18.5-25 kg/m^2^; Overweight: 25-30 kg/m^2^; Obese: 30+ kg/m^2^).^7^ Occupation was categorised as nurses (including student nurses and midwives); doctors and allied health professionals (i.e. other clinical roles requiring a higher degree, including medical students, pharmacists, physiotherapists, occupational therapists, etc.); clinical support roles (roles not requiring a higher degree, for example community health workers, housekeeping, or nursing assistants) and ‘other’ (comprising administrative and clerical staff, security and police).

Table A: Definitions used for HIV, hypertension, diabetes, mental health and multimorbidity in this study.

| **Condition** | **Definition of disease status** | **Definition of treated / controlled disease** |
| --- | --- | --- |
| HIV | ‘Known’ HIV positive if: a) self-report of previous positive HIV test and/or b) self-report of being on ART.  ‘Newly diagnosed’ HIV if positive HIV test result at ICAROZ.  HIV negative if: a) self-report of negative HIV test within the last 3 months or b) negative HIV test result at ICAROZ. | Considered ‘treated’ if known HIV and client reported being on ART. |
| Elevated BP | Known hypertension if a) self-reported diagnosis of hypertension and/or b) self-report of being on anti-hypertensive medications.  ‘Newly diagnosed’ elevated BP if a) systolic BP ≥140mmHg or b) diastolic BP ≥90mmHg on testing at ICAROZ. | Considered ‘controlled’ if known hypertension with systolic BP <140mmHg and diastolic BP <90mmHg at ICAROZ |
| Elevated HbA1c | Known diabetes if a) self-reported diagnosis of diabetes or b) self-report of being on anti-diabetic medications.  ‘Newly diagnosed’ elevated HbA1c if ≥6.5% on testing at ICAROZ. | Considered ‘controlled’ if known diabetes with HbA1c <6.5% at ICAROZ. |
| Common mental disorders | Known mental health condition if self-reported history of anxiety or depression.  ‘Newly diagnosed’ common mental disorder if SSQ score >8 at ICAROZ. | Considered ‘controlled’ if known mental health condition with SSQ score ≤8 at ICAROZ. |
| Multimorbidity | 2 or more of the chronic conditions as defined above. |  |
| **Abbreviations**:  ART = anti-retroviral therapy  BP = blood pressure  ICAROZ = Impact of the COVID-19 pandemic on health care workers and the health care system in Zimbabwe  SSQ = Shona symptom questionnaire | | |

## Multiple imputation

Multiple imputation by chained equations was performed in Stata (version 17), with 50 imputed datasets created. HIV was imputed as a binary variable (logistic regression). HbA1c was imputed as a continuous, non-normally distributed variable using predictive mean matching (PMM) the 10 closest subjects, and then categorised as described in Table 1.1.^8^ Covariates predicting missingness of HIV or HbA1c were included as described in Table 1.2. Clients in whom HIV testing was not indicated (known HIV positive or those who reported to have tested HIV negative in past 3 months) were excluded from the dataset for imputation of HIV test results; imputation of missing HbA1c in this group was performed separately and datasets combined.

Table B: Variables used in imputation procedure for HIV test results and HbA1c

|  | **Model / Form** |
| --- | --- |
| **Imputed variables** |  |
| HIV | Binary (logistic regression) |
| HbA1c | Continuous (PMM, 10 nearest) |
| **Covariates** |  |
| Sex | Binary |
| Age | Restricted cubic splines (5 knots) |
| Facility type / province | Categorical (11 categories) |
| Occupation | Categorical (4 categories)* |
| BMI | Categorical (4 categories) |
| Medical aid | Binary |
| Diastolic BP | Restricted cubic splines (5 knots) |
| Previous HIV test (self-reported) | Binary |
| Known diabetes | Binary |
| Known HIV | Binary |
| HIV testing (levels: onsite, offsite, declined) | Categorical |
| ***** Occupation was categorised as ‘support roles’ (not requiring any formal qualifications); nurses (including student nurses and midwives); doctors and allied health professionals (i.e. other clinical roles requiring a higher degree, including medical students, pharmacists, physiotherapists, occupational therapists, etc.); clinical support roles (roles not requiring a higher degree, for example community health workers, housekeeping, or nursing assistants) and ‘other’ (comprising administrative and clerical staff, security and police).  **Abbreviations**:  BMI = body mass index,  BP = blood pressure  PMM = predictive mean matching | |

In order to explore the potential of HIV test results being missing not at random, sensitivity analyses was conducted using the same model as used for the main imputation refit using the *offset* option within *mi estimate* for HIV status with offranging from -2 to +2. Here the offset is the difference in log odds of a positive HIV status among those with unobserved as compared to observed HIV status.

Imputation of SSQ scores for people who completed the paper-based questionnaire, to transform the distribution to resemble that obtained using ACASI, was performed separately to the above and included only the observed ACASI score as a potential predictor. Given the non-normal distribution of ACASI scores, the propensity mean matching method was used.

## Sample size calculation

Sample size calculations were not performed in advance. Illustrative sample sizes required to describe a prevalence with a range of precision are shown in the table. These suggested that this study had sufficient size to describe a condition with 20% prevalence with +/-1% precision. For conditions with lower prevalence than that, precision will be higher, for conditions with higher prevalence, precision will be lower.

Table C: Illustrative sample sizes to estimate a prevalence

| **95%CI +/-** | | 1% | 2% | 3% | 4% |
| --- | --- | --- | --- | --- | --- |
|  |  |  | **N required** | | |
| **Prevalence** | **1%** | 381 | 96 | 43 | 24 |
|  | **2%** | 753 | 189 | 84 | 48 |
|  | **5%** | 1825 | 457 | 203 | 115 |
|  | **10%** | 3458 | 865 | 385 | 217 |
|  | **20%** | 6147 | 1537 | 683 | 385 |
|  | **30%** | 8068 | 2017 | 897 | 505 |
|  | **40%** | 9220 | 2305 | 1025 | 577 |

# SUPPLEMENTARY RESULTS

## Uptake of disease screening and missing data

Table D: Uptake and results of screening for diabetes, HIV, hypertension and common mental disorders among healthcare workers in Zimbabwe (N=6598).

| **Condition** | **Screen negative** | **Screen positive** | **Known disease** | **Unknown*** |
| --- | --- | --- | --- | --- |
| **HIV** | 51% (3371) | 0% (17) | 10% (670) | 38% (2535) |
| **Elevated BP** | 64% (4222) | 19% (1248) | 17% (1123) | 0% (0) |
| **Elevated HbA1c** | 74% (4862) | 6.5% (428) | 3.6% (238) | 16% (1065) |
| **Common mental disorders** | 90% (5941) | 10% (645) | 0.1% (7) | 0% (0) |
| * Among 2535 people with unknown HIV status, 922 opted for offsite HIV self-testing (for which results are not known) and 1613 declined HIV testing. Data were not collected on the number of people who declined HbA1c testing, process evaluation (interviews with service providers) and interrogation of the patterns of missingness suggest that most of these missing data represent stock outs of test kits during the programme. | | | | |

Figure A: Uptake and results of screening for diabetes, HIV, hypertension and common mental disorders among healthcare workers in Zimbabwe (N=6598).


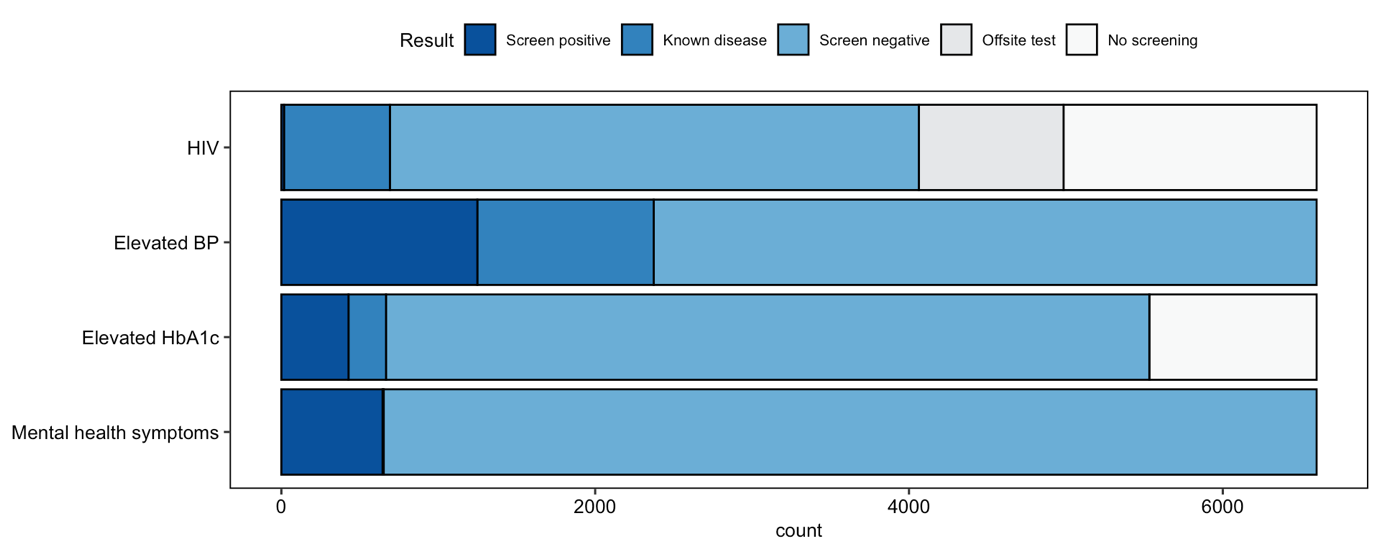


**Footnotes:** ‘No screening’ might be due to several reasons. Process evaluation of the project suggested that, for HIV, most people who were not screened did not want to get tested. For diabetes, this reflects stock outs of test kits. Participants who had tested negative for HIV in the previous 3 months (based on self-report) were not offered testing and are considered “screen negative” in the figure.

Figure B: Missing data for HIV test results and HbA1c, arranged by date (N = 6598).


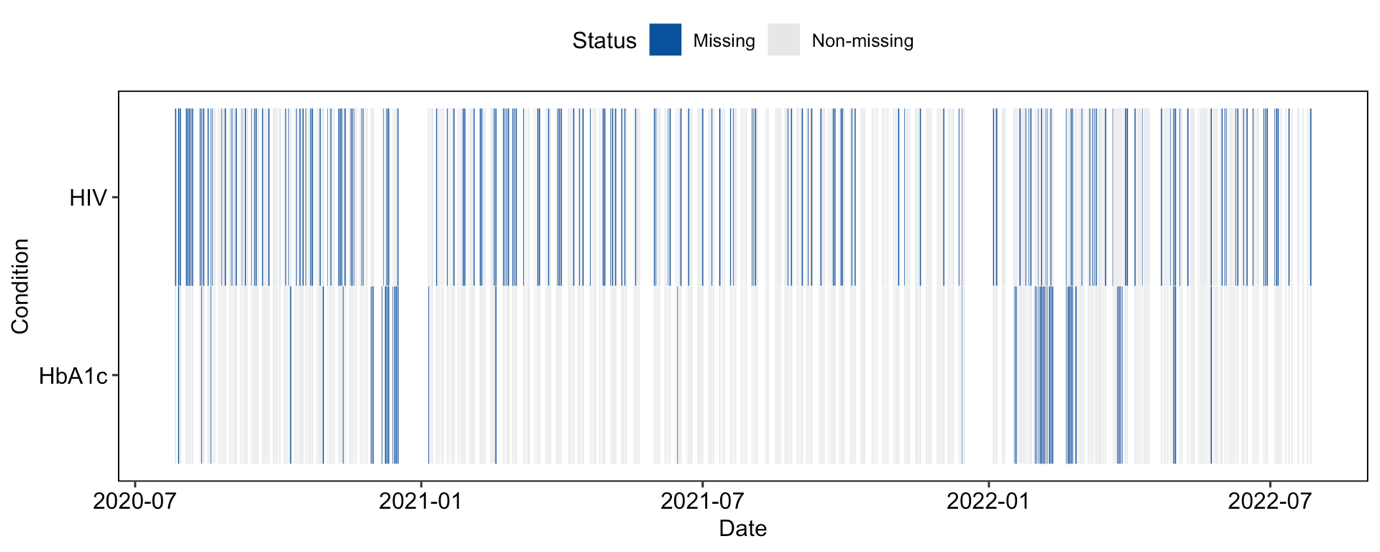


Table E: Distribution of missing data for HIV status among healthcare workers accessing the ICAROZ service (N = 6598).

|  |  | **HIV status known** | | **HIV status not known** | |
| --- | --- | --- | --- | --- | --- |
|  | **Overall** | **Already diagnosed with HIV or tested negative in past 3 months** | **Tested onsite** | **Tested offsite** | **HIV test not done** |
| **N** | 6598 | 1024 | 3039 | 922 | 1613 |
| **Sex** |  |  |  |  |  |
| Female | 5215 | 840 (16%) | 2,361 (45%) | 692 (13%) | 1,322 (25%) |
| Male | 1383 | 184 (13%) | 678 (49%) | 230 (17%) | 291 (21%) |
| **Age, years** | 37 (18-78) | 43 (18-76) | 36 (18-71) | 33 (18-65) | 37 (18-78) |
| **Province** |  |  |  |  |  |
| Harare | 4358 | 683 (16%) | 1,947 (45%) | 486 (11%) | 1,242 (28%) |
| Bulawayo | 516 | 56 (11%) | 261 (51%) | 150 (29%) | 49 (9.5%) |
| Mashonaland Central | 360 | 105 (29%) | 129 (36%) | 19 (5.3%) | 107 (30%) |
| Mashonaland East | 264 | 24 (9.1%) | 140 (53%) | 32 (12%) | 68 (26%) |
| Mashonaland West | 276 | 59 (21%) | 132 (48%) | 29 (11%) | 56 (20%) |
| Manicaland | 160 | 20 (12%) | 81 (51%) | 39 (24%) | 20 (12%) |
| Masvingo | 165 | 22 (13%) | 89 (54%) | 31 (19%) | 23 (14%) |
| Matabeleland South | 333 | 43 (13%) | 182 (55%) | 85 (26%) | 23 (6.9%) |
| Midlands | 166 | 12 (7.2%) | 78 (47%) | 51 (31%) | 25 (15%) |
| **Employer** |  |  |  |  |  |
| Government sector | 5,713 | 855 (15%) | 2,637 (46%) | 775 (14%) | 1,446 (25%) |
| Private sector | 338 | 32 (9.5%) | 184 (54%) | 76 (22%) | 46 (14%) |
| NGO/Other | 547 | 137 (25%) | 218 (40%) | 71 (13%) | 121 (22%) |
| **Occupation** |  |  |  |  |  |
| Support roles | 2576 | 606 (24%) | 1,063 (41%) | 232 (9.0%) | 675 (26%) |
| Nursing | 2754 | 300 (11%) | 1,349 (49%) | 505 (18%) | 600 (22%) |
| Doctors & AHP | 593 | 45 (7.6%) | 296 (50%) | 107 (18%) | 145 (24%) |
| Other | 675 | 73 (11%) | 331 (49%) | 78 (12%) | 193 (29%) |
| **Current smoker** | 175 | 30 (17%) | 77 (44%) | 26 (15%) | 42 (24%) |
| **Medical aid cover** | 4283 | 623 (15%) | 2,044 (48%) | 567 (13%) | 1,049 (24%) |
| **BMI, kg/m^2^** |  |  |  |  |  |
| <18.5, underweight | 171 | 25 (15%) | 83 (49%) | 31 (18%) | 32 (19%) |
| 18.5-24.9, healthy weight | 2259 | 356 (16%) | 987 (44%) | 378 (17%) | 538 (24%) |
| 25.0-29.9, overweight | 2036 | 315 (15%) | 956 (47%) | 261 (13%) | 504 (25%) |
| >30.0, obese | 2132 | 328 (15%) | 1,013 (48%) | 252 (12%) | 539 (25%) |
| **At least one known medical condition** | 1890 | 733 (39%) | 634 (34%) | 144 (7.6%) | 379 (20%) |
| **Known hypertension** | 1124 | 204 (18%) | 502 (45%) | 112 (10.0%) | 306 (27%) |
| **Known diabetes** | 239 | 39 (16%) | 105 (44%) | 27 (11%) | 68 (28%) |
| **Known mental health condition** | 7 | 4 (57%) | 2 (29%) | 1 (14%) | 0 (0%) |
| **Previous TB** | 2 | 2 (100%) | 0 (0%) | 0 (0%) | 0 (0%) |
| **Footnotes:** Test results were not known for people who opted for offsite testing.  **Abbreviations**:  AHP = allied health professionals  BMI = body mass index  N = number  NGO = non-governmental organisation  TB = tuberculosis | | | | | |

Table F: Distribution of missing data for HbA1c among study participants (N = 6598).

| **Characteristic** | **Overall** | **HbA1c known** | **HbA1c not known** |
| --- | --- | --- | --- |
| **N** | **6598** | **5499** | **1099** |
| **Sex** |  |  |  |
| Female | 5,215 (100%) | 4,353 (83%) | 862 (17%) |
| Male | 1,383 (100%) | 1,146 (83%) | 237 (17%) |
| **Age, years** | 37 (18-78) | 37 (18-78) | 35 (18-65) |
| **Province** |  |  |  |
| Harare | 4,358 (100%) | 3,966 (91%) | 392 (9.0%) |
| Bulawayo | 516 (100%) | 154 (30%) | 362 (70%) |
| Mashonaland Central | 360 (100%) | 356 (99%) | 4 (1.1%) |
| Mashonaland East | 264 (100%) | 76 (29%) | 188 (71%) |
| Mashonaland West | 276 (100%) | 274 (99%) | 2 (0.7%) |
| Manicaland | 160 (100%) | 128 (80%) | 32 (20%) |
| Masvingo | 165 (100%) | 164 (99%) | 1 (0.6%) |
| Matabeleland South | 333 (100%) | 247 (74%) | 86 (26%) |
| Midlands | 166 (100%) | 134 (81%) | 32 (19%) |
| **Employer** |  |  |  |
| Government sector | 5,713 (100%) | 4,631 (81%) | 1,082 (19%) |
| Private sector | 338 (100%) | 333 (99%) | 5 (1.5%) |
| NGO/Other | 547 (100%) | 535 (98%) | 12 (2.2%) |
| **Occupation** |  |  |  |
| Support roles | 2,576 (100%) | 2,161 (84%) | 415 (16%) |
| Nursing | 2,754 (100%) | 2,255 (82%) | 499 (18%) |
| Doctors & AHP | 593 (100%) | 520 (88%) | 73 (12%) |
| Other | 675 (100%) | 563 (83%) | 112 (17%) |
| **Current smoker** | 175 (100%) | 148 (85%) | 27 (15%) |
| **Medical aid cover** | 4,283 (100%) | 3,614 (84%) | 669 (16%) |
| **BMI, kg/m2** |  |  |  |
| <18.5, underweight | 171 (100%) | 134 (78%) | 37 (22%) |
| 18.5-24.9, healthy | 2,259 (100%) | 1,856 (82%) | 403 (18%) |
| 25.0-29.9, overweight | 2,036 (100%) | 1,714 (84%) | 322 (16%) |
| >30.0, obese | 2,132 (100%) | 1,795 (84%) | 337 (16%) |
| **At least one known medical condition** | 1,890 (100%) | 1,623 (86%) | 267 (14%) |
| **Known HIV** | 675 (100%) | 567 (84%) | 108 (16%) |
| **On ART** | 670 (100%) | 563 (84%) | 107 (16%) |
| **Known hypertension** | 1,124 (100%) | 966 (86%) | 158 (14%) |
| **Known diabetes** | 239 (100%) | 206 (86%) | 33 (14%) |
| **Known mental health condition** | 7 (100%) | 6 (86%) | 1 (14%) |
| **Previous TB** | 2 (100%) | 2 (100%) | 0 (0%) |
| **Abbreviations**:  AHP = allied health professionals  BMI = body mass index  N = number  NGO = non-governmental organisation  TB = tuberculosis | | | |

Table G: Distribution of covariates by SSQ format among healthcare workers accessing the ICAROZ service (N = 6598).

| **Characteristic** | **Overall** | **Completed SSQ using paper questionnaire** | **Completed SSQ using ACASI** |
| --- | --- | --- | --- |
| **N** | **6598** | **4603 (70%)** | **1995 (30%)** |
| **N with SSQ>8** | 646 | 383 (59%) | 263 (41%) |
| **Sex** |  |  |  |
| Female | 5,215 (100%) | 3,653 (70%) | 1,562 (30%) |
| Male | 1,383 (100%) | 950 (69%) | 433 (31%) |
| **Age, years** | 37 (18-78) | 37 (18-76) | 37 (18-78) |
| **Province** |  |  |  |
| Harare | 4,358 (100%) | 3,759 (86%) | 599 (14%) |
| Bulawayo | 516 (100%) | 62 (12%) | 454 (88%) |
| Mashonaland Central | 360 (100%) | 298 (83%) | 62 (17%) |
| Mashonaland East | 264 (100%) | 0 (0%) | 264 (100%) |
| Mashonaland West | 276 (100%) | 69 (25%) | 207 (75%) |
| Manicaland | 160 (100%) | 68 (42%) | 92 (57%) |
| Masvingo | 165 (100%) | 115 (70%) | 50 (30%) |
| Matabeleland South | 333 (100%) | 197 (59%) | 136 (41%) |
| Midlands | 166 (100%) | 35 (21%) | 131 (79%) |
| **Employer** |  |  |  |
| Government sector | 5,713 (100%) | 4,091 (72%) | 1,622 (28%) |
| Private sector | 338 (100%) | 218 (64%) | 120 (36%) |
| NGO/Other | 547 (100%) | 294 (54%) | 253 (46%) |
| **Occupation** |  |  |  |
| Support roles | 2,576 (100%) | 1,761 (68%) | 815 (32%) |
| Nursing | 2,754 (100%) | 1,919 (70%) | 835 (30%) |
| Doctors & AHP | 593 (100%) | 434 (73%) | 159 (27%) |
| Other | 675 (100%) | 489 (72%) | 186 (28%) |
| **Current smoker** | 175 (100%) | 118 (67%) | 57 (33%) |
| **Medical aid cover** | 4,283 (100%) | 3,073 (72%) | 1,210 (28%) |
| **BMI category** |  |  |  |
| <18.5 kg/m^2^, Underweight | 171 (100%) | 116 (68%) | 55 (32%) |
| 18.5–24.9 kg/m^2^, Healthy BMI | 2,259 (100%) | 1,605 (71%) | 654 (29%) |
| 25.0­–29.9 kg/m^2^, Overweight | 2,036 (100%) | 1,430 (70%) | 606 (30%) |
| >30.0 kg/m^2^, Obese | 2,132 (100%) | 1,452 (68%) | 680 (32%) |
| **At least one known medical condition** | 1,890 (100%) | 1,284 (68%) | 606 (32%) |
| **Known hypertension** | 1,124 (100%) | 759 (68%) | 365 (32%) |
| **Known diabetes** | 239 (100%) | 167 (70%) | 72 (30%) |
| **Known mental health condition** | 7 (100%) | 4 (57%) | 3 (43%) |
| **Previous TB** | 2 (100%) | 1 (50%) | 1 (50%) |
| **Abbreviations**:  ACASI= Audio Computer-Assisted Self Interviewing  AHP = allied health professionals  BMI = body mass index  N = number  NGO = non-governmental organisation  SSQ = Shona Symptom Questionnaire  TB = tuberculosis | | | |

Table H: Distribution of observed and imputed values of HIV status and HbA1c across original and 50 imputed datasets.

|  | **HIV status**  **N (%)** | | | **HbA1c** | | **Diabetes status**  **N (%)** | | |
| --- | --- | --- | --- | --- | --- | --- | --- | --- |
| **Dataset** | **None** | **Known** | **Newly diagnosed** | **Mean** | **Std. dev** | **None** | **Known** | **Newly diagnosed** |
| Original* | 3371 (83%) | 675 (17%) | 17 (0.4%) | 5.72 | 0.96 | 4865 (88%) | 239 (4.3%) | 428 (7.7%) |
| Across 50 imputed datasets | 5862 (89%) | 675 (10%) | 60.9 (0.9%) | 5.74 | 0.95 | 5818 (89%) | 239 (3.6%) | 541 (8.2%) |
| **Footnotes:**  *2535 missing HIV status in the original dataset. 1065 missing HbA1c in the original dataset.  **Abbreviations**:  N = number, % = percentage | | | | | | | | |

Table I: Distribution of observed and imputed values of SSQ

|  | **SSQ** | | **Common mental disorders (SSQ >8) status**  **N (%)** | | |
| --- | --- | --- | --- | --- | --- |
| **Dataset** | **Mean** | **Std. dev** | **None** | **Known** | **Newly diagnosed** |
| Original | 3.89 | 3.06 | 5946 (90%) | 7 (0.2%) | 705 (10.7%) |
| Across 50 imputed datasets* | 4.44 | 2.92 | 5891 (89%) | 7 (0.2%) | 541 (9.7%) |
| **Footnotes**:  * Values imputed for 4603 who completed an audio computer-assisted self-interviewing (ACASI) Shona Symptom Questionnaire (SSQ) in the original dataset.  **Abbreviations**:  N = number, Std. dev = standard deviation, % = percentage. | | | | | |

## Prevalence of chronic disease stratified by age and BMI category

Table J: Prevalence of chronic diseases and multimorbidity by age (in multiply imputed dataset)

|  |  | **% prevalence (95% confidence interval)** | | |
| --- | --- | --- | --- | --- |
| **Condition** | **Age, years** | **Overall** | **Known** | **Screening detected** |
| **Multimorbidity** | 18–29 | 3.6 (2.6–4.7) | **–** | **–** |
|  | 30–39 | 8.6 (7.0–10.1) | **–** | **–** |
|  | 40–49 | 20.9 (17.6–24.2) | **–** | **–** |
|  | 50+ | 39.4 (35.7–43.0) | **–** | **–** |
| **HIV** | 18–29 | 5.0 (2.6–7.3) | 4.0 (1.9–6.2) | 0.9 (0.1–1.8) |
|  | 30–39 | 6.5 (5.2–7.9) | 5.9 (4.6–7.2) | 0.6 (0.0–1.3) |
|  | 40–49 | 15.4 (12.4–18.4) | 14.4 (11.5–17.4) | 1.0 (0.2–1.8) |
|  | 50+ | 25.7 (20.9–30.5) | 24.2 (19.5–28.9) | 1.5 (0.3–2.6) |
| **Elevated BP** | 18–29 | 13.1 (10.5–15.8) | 1.0 (0.4–1.6) | 12.2 (9.3–15.0) |
|  | 30–39 | 30.5 (25.5–35.4) | 10.6 (8.3–13.0) | 19.8 (15.1–24.6) |
|  | 40–49 | 47.7 (44.6–50.8) | 24.5 (21.7–27.3) | 23.3 (19.2–27.3) |
|  | 50+ | 69.4 (65.9–72.9) | 47.8 (44.1–51.5) | 21.6 (18.9–24.4) |
| **Elevated HbA1c** | 18–29 | 5.2 (3.7–6.7) | 0.4 (0.1–0.7) | 4.8 (3.3–6.4) |
|  | 30–39 | 8.1 (6.1–10.1) | 1.6 (0.8–2.4) | 6.5 (4.8–8.2) |
|  | 40–49 | 15.3 (12.8–17.8) | 5.0 (3.2–6.7) | 10.3 (8.5–12.2) |
|  | 50+ | 26.7 (22.8–30.6) | 11.7 (9.8–13.5) | 15.0 (11.3–18.6) |
| **Common mental disorders** | 18–29 | 10.4 (8.0–12.8) | 0.1 (-0.1–0.2) | 9.2 (6.2–12.2) |
|  | 30–39 | 10.0 (7.8–12.2) | 0.1 (0.0–0.3) | 8.2 (5.9–10.4) |
|  | 40–49 | 11.4 (8.8–14.0) | 0.1 (-0.1–0.3) | 11.9 (8.7–15.1) |
|  | 50+ | 11.8 (8.4–15.2) | 0.1 (-0.1–0.3) | 11.0 (7.7–14.2) |
| **Footnotes:**  Clients were considered to have a condition if they either self-reported or screened positive during the study. Screening positive was defined as follows. Elevated blood pressure (BP): systolic BP ≥140mmHg or diastolic BP ≥90mmgHg. Elevated HbA1c: ≥6.5%. Mental health symptoms: Shona Symptom Questionnaire Score >8. Multimorbidity was defined as having two or more of the chronic conditions listed in the table. Estimates are adjusted for health facility-level clustering using robust standard errors. | | | | |

Table K: Prevalence of chronic diseases and multimorbidity by BMI category (in multiply imputed dataset)

|  | **% Prevalence (95%CI)** | | | |
| --- | --- | --- | --- | --- |
| **Condition** | **Underweight** | **Healthy BMI** | **Overweight** | **Obese** |
| Multimorbidity | 9.5 (4.8–14.2) | 9.3 (7.4–11.3) | 14.3 (11.6–16.9) | 21.8 (18.4–25.2) |
| HIV | 11.7 (5.2–18.3) | 11.7 (8.5–14.9) | 11.1 (7.9–14.4) | 10.5 (8.5–12.6) |
| Elevated BP | 19.3 (14.0–24.6) | 23.9 (20.2–27.6) | 35.4 (31.3–39.5) | 50.6 (48.2–53.0) |
| Elevated HbA1c | 5.8 (1.3–10.3) | 6.9 (5.5–8.2) | 11.6 (9.6–13.6) | 18.0 (14.6–21.3) |
| Common mental disorders | 11.5 (5.2–17.8) | 10.7 (8.5–12.9) | 10.7 (8.1–13.2) | 10.7 (8.6–12.7) |
| **Footnotes:**  Clients were considered to have a condition if they either self-reported or screened positive during the study. Screening positive was defined as follows. Elevated blood pressure (BP): systolic BP ≥140mmHg or diastolic BP ≥90mmgHg. Elevated HbA1c: ≥6.5%. Common mental disorders: Shona Symptom Questionnaire Score >8. Multimorbidity was defined as having two or more of the chronic conditions listed in the table. BMI was categorized according to WHO definitions: Underweight: <18.5 kg/m^2^; Healthy BMI: 18.5-25 kg/m^2^; Overweight: 25-30 kg/m^2^; Obese: 30+ kg/m^2^. Estimates are adjusted for health facility-level clustering using robust standard errors. | | | | |

## Prevalence of multimorbidity by different definitions

Table L: Prevalence of multimorbidity by different definitions

|  | **N conditions *** | **% prevalence (95% CI)** |
| --- | --- | --- |
| **All conditions** | ≥1 condition | 52.6 (50.2–54.9) |
|  | ≥2 conditions | 14.9 (12.7–17.1) |
|  | ≥3 conditions | 2.1 (1.5–2.7) |
| **Newly screening positive** | ≥1 new condition | 34.0 (31.4–36.6) |
|  | ≥2 new conditions | 4.6 (3.9–5.4) |
| **Known conditions** | ≥2 known conditions | 5.8 (4.4–7.2) |
| **Footnotes**: * Included conditions: diabetes, hypertension, HIV, common mental disorders. Estimates are adjusted for health facility-level clustering using robust standard errors.  **Abbreviations**: 95%CI = 95% confidence interval | | |

## Classification of disease severity for blood pressure and HbA1c

Table M: Classification of blood pressure

|  | **In multiply imputed dataset*** | | |
| --- | --- | --- | --- |
|  | **Overall** | **Known** | **Screening detected** |
| Normal BP | 71% (67–74) | 39% (34–44%) | ­– |
| Mildly elevated BP | 19% (17–21) | 34% (31–37%) | 72% (69–75%) |
| Moderately elevated BP | 6.6% (5.5–7.6) | 17% (14–20%) | 20% (17–22%) |
| Severely elevated BP | 3.4% (2.5­–4.2) | 10% (7.5–12%) | 8.8% (6.9–11%) |
| *Median SBP, mmHg* | *123 (123–123)* | *135 (134–136)* | *141 (140–142)* |
| *Median DBP, mmHg* | *82 (82–82)* | *90 (89–91)* | *94 (94–94)* |
| **Footnotes**: * % Presented as prevalence (95% confidence interval) or median (95% confidence interval) estimated using mi estimate: proportion and mi estimate: qreg {var}, quantile(50). Blood pressure categories are defined with reference to WHO hypertension categories: mildy elevated BP = systolic (SBP) 140-159 millimetres of mercury (mmHg) or diastolic (DBP) 90­–99mmHg (stage 1 hypertension); moderately elevated BP = systolic 160­–179mmHg or diastolic 100­–109 (stage 2 hypertension); severely elevated BP = systolic ≥180mmHg or diastolic ≥110mmHg (stage 3 hypertension). Estimates are adjusted for health facility-level clustering using robust standard errors. | | | |

Table N: Classification of HbA1c

|  | **In multiply imputed dataset*** | | | |
| --- | --- | --- | --- | --- |
| **HbA1c category** | **Overall** | **No diabetes** | **Known diabetes** | **Diabetes detected by screening** |
| <6% | 70% (65­–76%) | 79% (74–83%) | 27% (19–36%) | – |
| 6–6.4% | 19% (16–23%) | 21% (17–26%) | 18% (14–22%) | – |
| 6.5–6.9% | 5.7% (4.5–6.8%) | – | 13% (8.6–18%) | 63% (58–68%) |
| 7–7.9% | 2.1% (1.5–2.7%) | – | 11% (6.4–15%) | 21% (17–25%) |
| ≥8% | 2.4% (1.9–2.9%) | – | 30% (23–37%) | 16% (11–21%) |
| *Median HbA1c, %* | *5.6% (5.6–5.6%)* | *5.5% (5.5–­5.5%)* | *6.6% (6.3–6.9%)* | *6.8% (6.7–6.9%)* |
| **Footnotes**: * % Presented as prevalence (95% confidence interval) or median (95% confidence interval) estimated using mi estimate: proportion and mi estimate: qreg {var}, quantile(50). Adjusted for health facility-level clustering using robust standard errors. | | | | |

## Disease control status for chronic conditions

Table O: HIV treatment care cascade (N = 6593)

|  | **% prevalence (95% CI)** | | |
| --- | --- | --- | --- |
| **Subgroup** | **Total, known PLHIV** | **Known PLHIV, on treatment** | **Known PLHIV, not on treatment** |
| **OVERALL** | 10.2 (7.7–12.7) | 10.2 (7.7–12.6) | 0.07 (0.0–0.1) |
| **WOMEN** | 10.7 (8.2–13.3) | 10.6 (8.1–13.1) | 0.1 (0.0–0.2) |
| **MEN** | 8.4 (5.5–11.3) | 8.4 (5.5–11.3) | 0.0 (0.0–0.0) |
| **Abbreviations:** PLHIV = people living with HIV | | | |

Table P: Disease control status for chronic conditions, in multiply imputed dataset (N = 6593)

|  | **% prevalence (95% CI)** | | |
| --- | --- | --- | --- |
|  | **Total, known** | **Known, controlled** | **Known, uncontrolled** |
| **OVERALL** | | | |
| Elevated BP | 17.0 (14.9–19.1) | 6.7 (5.2–8.1) | 10.4 (9.3–11.4) |
| Elevated HbA1c | 3.6 (2.8–4.4) | 1.7 (1.1–2.3) | 1.9 (1.5–2.4) |
| Common mental disorders | 0.2 (0.1–0.5) | 0.1 (0.0–0.2) | 0.0 (0.0–0.0) |
| **WOMEN** | | | |
| Elevated BP | 19.0 (16.5–21.5) | 7.6 (5.8–9.4) | 11.4 (10.2–12.6) |
| Elevated HbA1c | 4.1 (3.2–4.9) | 1.9 (1.2–2.6) | 2.2 (1.7–2.6) |
| Common mental disorders | 0.1 (0.0–0.2) | 0.1 (0.0–0.1) | 0.0 (0.0–0.1) |
| **MEN** | | | |
| Elevated BP | 9.7 (7.8–11.6) | 3.3 (2.4–4.3) | 6.4 (4.8–8.0) |
| Elevated HbA1c | 1.9 (0.8–2.9) | 0.8 (0.2–1.4) | 1.1 (0.4–1.7) |
| Common mental disorders | 0.2 (0.0–0.5) | 0.2 (-0.1–0.5) | 0.0 (0.0–0.0) |

Table Q: Multimorbidity disease control status, in multiply imputed dataset (N = 6593)

| **Outcome** | **% prevalence (95% CI)** |
| --- | --- |
| One controlled/treated disease | 8.6 (7.1–10.1) |
| One uncontrolled disease | 29.1 (26.2–31.9) |
| Two or more controlled/treated diseases | 1.0 (0.7–1.4) |
| Two or more diseases, one uncontrolled | 6.1 (4.6–7.6) |
| Two or more uncontrolled diseases | 7.8 (6.7–8.8) |

## Disease patterns among people with multimorbidity

Among people with multimorbidity (2 or more conditions), 82% were women and the median age was 46 years (IQR 39–54). The prevalence of individual conditions is shown in table 2.15.

Table R: Disease prevalence among people with multimorbidity

| **Condition** | **Overall** | **Men** | **Women** |
| --- | --- | --- | --- |
| Elevated HbA1c | 53% | 49% | 54% |
| HIV | 39% | 39% | 39% |
| Elevated blood pressure | 88% | 91% | 87% |
| Mental health symptoms | 36% | 37% | 36% |

Among people with multimorbidity most common disease combinations were:

- Hypertension and diabetes (46%)
- Hypertension and HIV (30%)
- Hypertension and mental health (27%)

The most common triad was hypertension, HIV and diabetes (4% of people with multimorbidity).

## Risk factors for chronic conditions and multimorbidity

Table S: Associations with elevated blood pressure, elevated HbA1c and multimorbidity in a fully-adjusted multivariable model (including body mass index), after multiple imputation.

| **Variable** | **Level** | **aOR (95%CI)*** | **p** |
| --- | --- | --- | --- |
| **MULTIMORBIDITY** | | | |
| **Sex** | Women | – | 0.2 |
|  | Men | 0.84 (0.65–1.09) |  |
| **Age, years** | 18–29 | – | <0.001* |
|  | 30–39 | 2.15 (1.57–2.94) |  |
|  | 40–49 | 5.67 (4.17–7.70) |  |
|  | 50+ | 13.80 (10.14–18.79) |  |
| **Occupation** | Support roles | – | 0.02 |
|  | Nursing | 0.72 (0.56–0.91) |  |
|  | Doctors & AHP | 0.70 (0.48–1.02) |  |
|  | Other | 0.73 (0.56–0.94) |  |
| **BMI category** | <18.5 kg/m^2^, Underweight | 1.40 (0.81–2.42) | 0.04* |
|  | 18.5–24.9 kg/m^2^, Healthy BMI | – |  |
|  | 25.0­–29.9 kg/m^2^, Overweight | 1.21 (0.97–1.51) |  |
|  | >30.0 kg/m^2^, Obese | 1.64 (1.27–2.12) |  |
| **ELEVATED BLOOD PRESSURE** | | | |
| **Sex** | Women | – | 0.007 |
|  | Men | 1.23 (1.06–1.43) |  |
| **Age, years** | 18–29 | – | <0.001* |
|  | 30–39 | 2.41 (1.99–2.92) |  |
|  | 40–49 | 4.98 (4.29–5.78) |  |
|  | 50+ | 12.21 (9.47–15.76) |  |
| **Occupation** | Support roles | – | 0.002 |
|  | Nursing | 1.19 (1.06–1.33) |  |
|  | Doctors & AHP | 1.23 (0.93–1.63) |  |
|  | Other | 1.26 (1.07–1.49) |  |
| **BMI category** | <18.5 kg/m^2^, Underweight | 0.94 (0.67–1.31) | <0.001* |
|  | 18.5–24.9 kg/m^2^, Healthy BMI | – |  |
|  | 25.0­–29.9 kg/m^2^, Overweight | 1.39 (1.16–1.67) |  |
|  | >30.0 kg/m^2^, Obese | 2.24 (1.90–2.64) |  |
| **ELEVATED HBA1C** | | | |
| **Sex** | Women | – | 0.9 |
|  | Men | 1.01 (0.77–1.33) |  |
| **Age, years** | 18–29 | – | <0.001* |
|  | 30–39 | 1.27 (0.96–1.68) |  |
|  | 40–49 | 2.52 (1.90–3.32) |  |
|  | 50+ | 4.94 (3.59–6.80) |  |
| **Occupation** | Support roles | – | 0.8 |
|  | Nursing | 11.03 (0.87–1.22) |  |
|  | Doctors & AHP | 0.92 (0.67–1.27) |  |
|  | Other | 1.11 (0.85–1.44) |  |
| **BMI category** | <18.5 kg/m^2^, Underweight | 0.94 (0.40–2.20) | 0.003* |
|  | 18.5–24.9 kg/m^2^, Healthy BMI | – |  |
|  | 25.0­–29.9 kg/m^2^, Overweight | 1.52 (1.22–1.89) |  |
|  | >30.0 kg/m^2^, Obese | 2.18 (1.71–2.76) |  |
| P value for association presented; * indicates that the p-value for linear trend was <0.001.  aOR is adjusted for variables shown in the table and adjusted for clinic level clustering using robust standard errors.  **Abbreviations:**  AHP = Allied health professional  BMI = body mass index | | | |

## Disease prevalence stratified by HIV status

Table T: Prevalence of chronic conditions stratified by HIV status

|  | **No HIV*** | | | **PLHIV*** | | | **OR†** | **aOR†** | **p (aOR)** |
| --- | --- | --- | --- | --- | --- | --- | --- | --- | --- |
| **Condition** | **Overall** | **Known** | **Screening detected** | **Overall** | **Known** | **Screening detected** |  |  |  |
| **Multi-morbidity‡** | **10.2**  **(8.7–11.7)** | **–** | **–** | **13.2**  **(10.3–16.0)** | **–** | **–** | 1.33  (1.02–1.73) | 0.74  (0.56–0.98) | 0.04 |
| Elevated BP | **35.5**  **(32.8–38.1)** | 16.6  (14.4–18.8) | 18.9  (15.2–22.5) | **40.0**  **(35.4–44.5)** | 20.5  (16.7–24.4) | 19.4  (15.9–23.0) | 1.21  (0.99–1.47) | 0.68  (0.55–0.84) | <0.001 |
| Elevated HbA1c | **11.8**  **(9.9–13.7)** | 3.6  (2.7–4.5) | 8.2  (6.7–9.7) | **12.9**  **(10.0–15.8)** | 3.8  (2.4–5.2) | 9.1  (6.3–12.0) | 1.11  (0.85–1.44) | 0.69  (0.52–0.89) | 0.006 |
| Common mental disorders | **10.3**  **(8.6–12.0)** | 0.1  (0.0–0.2) | 10.3  (8.6–12.0) | **13.9**  **(9.6–18.3)** | 0.1 (0.0–0.9) | 13.9  (9.6–18.2) | 1.41  (1.02–1.93) | 1.23  (0.92–1.65) | 0.2 |
| Health facility-level clustering was accounted for in all estimates using robust standard errors.  * Presented as % prevalence (95% confidence interval)  † Odds ratios are of being PLHIV, compared to people who were not living with HIV (reference group). aOR is adjusted for age (4-bands), sex and occupation. P values presented are from the adjusted model (aOR). Additional adjustment for BMI attenuated the relationship between HIV and multimorbidity (0.81, 95% CI 0.62–1.07), elevated BP (0.73; 95%CI 0.59–0.90) and elevated HbA1c (0.74; 95%CI 0.57–0.96).  ‡ Multimorbidity is defined here as two or more of elevated blood pressure, elevated HbA1c or symptoms of common mental disorders (Shona Symptom Questionnaire score >8).  **Abbreviations:**  95%CI = 95% confidence interval  (a)OR = (adjusted) odds ratio  PLHIV = people living with HIV. | | | | | | | | | |

Table U: Odds ratios for (a) known diabetes and (b) known hypertension among all healthcare workers with elevated HbA1c or elevated blood pressure (respectively), comparing people with HIV and on treatment (‘in care’) to people without HIV

|  | **n/N*** | **OR (95% CI)** | **aOR (95% CI)** † |
| --- | --- | --- | --- |
| 1. **Known diabetes (among people with elevated HbA1c)** | | | |
| No HIV | 213/589 | – | – |
| PLHIV in care | 26/78 | 0.88 (0.55–1.43) | 0.68 (0.43–1.06) |
| 1. **Known hypertension (among people with elevated BP)** | | | |
| No HIV | 989/2105 | – | – |
| PLHIV in care | 135/268 | 1.15 (0.85–1.55) | 0.78 (0.57–1.05) |
| **Footnotes**: * Number with known diabetes or known hypertension respectively (outcome; n) / number with respective level of exposure (HIV; N) using complete case numbers (N=3390).  † Adjusted for age, sex and occupation. Clustering was accounted for in all estimates using robust standard errors.  **Abbreviations**: BP = blood pressure, OR = odds ratio, aOR = adjusted OR, 95% CI = 95% confidence interval | | | |

##

## Prevalence of HIV and multimorbidity in sensitivity analysis

Table V: Prevalence of HIV and multimorbidity across different imputation settings, and complete case records approach

|  | **Original dataset**  **(N = 3390)*** | **MI datasets (N = 6598)*** | | | | |
| --- | --- | --- | --- | --- | --- | --- |
|  |  | **Main** | **Delta -2** | **Delta -1** | **Delta 1** | **Delta 2** |
| New HIV | 0.4  (0.3–0.7) | 0.9  (0.5–1.4) | 0.6  (0.2–0.9) | 0.7  (0.2–1.2) | 1.6  (0.7–2.4) | 2.8  (1.5–4.1) |
| Overall HIV | 17.2  (15.9–18.5) | 11.2  (8.6–13.7) | 10.8  (8.2–13.4) | 10.9  (8.4–13.5) | 11.8  (9.3–14.3) | 13.0  (10.5–15.6) |
| Multimorbidity | 18.1  (16.8–19.4) | 14.9  (12.7–17.1) | 14.5  (12.1–16.9) | 14.6  (12.2–17.0) | 14.9  (12.6–17.3) | 15.4  (13.1–17.7) |
| **Footnotes:** * Presented as % prevalence (95% confidence interval). Multiple imputation with delta of 1 (highlighted) reflects a scenario where the proportion of people with undiagnosed HIV in the study population is that described in the general Zimbabwean population (13% adults with unknown HIV status).^9^  **Abbreviations**: MI = multiply imputed | | | | | | |

# COMPLETED STROBE CHECKLIST

Table W: STROBE checklist

| Domain | Item No | Recommendation | Page N |
| --- | --- | --- | --- |
| Title and abstract | 1 | (*a*) Indicate the study’s design with a commonly used term in the title or the abstract | 1 |
|  |  | (*b*) Provide in the abstract an informative and balanced summary of what was done and what was found | 2 |
| Introduction | | |  |
| Background/  rationale | 2 | Explain the scientific background and rationale for the investigation being reported | 5 |
| Objectives | 3 | State specific objectives, including any prespecified hypotheses | 5 |
| Methods | | |  |
| Study design | 4 | Present key elements of study design early in the paper | 5 |
| Setting | 5 | Describe the setting, locations, and relevant dates, including periods of recruitment, exposure, follow-up, and data collection | 5 |
| Participants | 6 | (*a*) Give the eligibility criteria, and the sources and methods of selection of participants | 5 |
| Variables | 7 | Clearly define all outcomes, exposures, predictors, potential confounders, and effect modifiers. Give diagnostic criteria, if applicable | 6 |
| Data sources/ measurement | 8* | For each variable of interest, give sources of data and details of methods of assessment (measurement). Describe comparability of assessment methods if there is more than one group | 6 |
| Bias | 9 | Describe any efforts to address potential sources of bias | 6 |
| Study size | 10 | Explain how the study size was arrived at | 5 |
| Quantitative variables | 11 | Explain how quantitative variables were handled in the analyses. If applicable, describe which groupings were chosen and why | 6-7 |
| Statistical methods | 12 | (*a*) Describe all statistical methods, including those used to control for confounding | 6-7 |
|  |  | (*b*) Describe any methods used to examine subgroups and interactions | 6-7 |
|  |  | (*c*) Explain how missing data were addressed | 6 |
|  |  | (*d*) If applicable, describe analytical methods taking account of sampling strategy | 6 |
|  |  | (*e*) Describe any sensitivity analyses | 6 |
| Results | | |  |
| Participants | 13* | (a) Report numbers of individuals at each stage of study—eg numbers potentially eligible, examined for eligibility, confirmed eligible, included in the study, completing follow-up, and analysed | 7 |
|  |  | (b) Give reasons for non-participation at each stage | 7 |
|  |  | (c) Consider use of a flow diagram | NA |
| Descriptive data | 14* | (a) Give characteristics of study participants (eg demographic, clinical, social) and information on exposures and potential confounders | 8 |
|  |  | (b) Indicate number of participants with missing data for each variable of interest | 8 |
| Outcome data | 15* | Report numbers of outcome events or summary measures | 9 |
| Main results | 16 | (*a*) Give unadjusted estimates and, if applicable, confounder-adjusted estimates and their precision (eg, 95% confidence interval). Make clear which confounders were adjusted for and why they were included | 11 |
|  |  | (*b*) Report category boundaries when continuous variables were categorized | 8 |
|  |  | (*c*) If relevant, consider translating estimates of relative risk into absolute risk for a meaningful time period | NA |
| Other analyses | 17 | Report other analyses done—eg analyses of subgroups and interactions, and sensitivity analyses | 10 |
| Discussion | | |  |
| Key results | 18 | Summarise key results with reference to study objectives | Done |
| Limitations | 19 | Discuss limitations of the study, taking into account sources of potential bias or imprecision. Discuss both direction and magnitude of any potential bias | Done |
| Interpretation | 20 | Give a cautious overall interpretation of results considering objectives, limitations, multiplicity of analyses, results from similar studies, and other relevant evidence | Done |
| Generalisability | 21 | Discuss the generalisability (external validity) of the study results | Done |
| Other information | | |  |
| Funding | 22 | Give the source of funding and the role of the funders for the present study and, if applicable, for the original study on which the present article is based | Done |

# REFERENCES

1 Ministry of Health and Child Care. Zimbabwe national guidlines on HIV testing and counselling (second edition). Harare, Zimbabwe: Ministry of Health and Child Care, 2014 https://hivstar.lshtm.ac.uk/files/2016/06/ZIMBABWE-National-Guidlines-on-HTC-2014.compressed.pdf (accessed May 31, 2023).

2 Chibanda D, Verhey R, Gibson LJ, *et al.* Validation of screening tools for depression and anxiety disorders in a primary care population with high HIV prevalence in Zimbabwe. *J Affect Disord* 2016; **198**: 50–5.

3 World Health Organization. WHO consolidated guidelines on tuberculosis. Module 2: screening – systematic screening for tuberculosis disease. Geneva: World Health Organization, 2021.

4 Patel V, Simunyu E, Gwanzura F, Lewis G, Mann A. The Shona Symptom Questionnaire: The development of an indigenous measure of common mental disorders in Harare. *Acta Psychiatr Scand* 1997. DOI:10.1111/j.1600-0447.1997.tb10134.x.

5 Price AJ, Crampin AC, Amberbir A, *et al.* Prevalence of obesity, hypertension, and diabetes, and cascade of care in sub-Saharan Africa: a cross-sectional, population-based study in rural and urban Malawi. *Lancet Diabetes Endocrinol* 2018; **6**: 208–22.

6 Wong EB, Olivier S, Gunda R, *et al.* Convergence of infectious and non-communicable disease epidemics in rural South Africa: a cross-sectional, population-based multimorbidity study. *Lancet Glob Health* 2021; **9**: e967–76.

7 Obesity and overweight. https://www.who.int/news-room/fact-sheets/detail/obesity-and-overweight (accessed March 26, 2023).

8 Morris TP, White IR, Royston P. Tuning multiple imputation by predictive mean matching and local residual draws. *BMC Med Res Methodol* 2014; **14**: 75.

9 Ministry of Health and Child Care (MOHCC). Zimbabwe Population-based HIV impact assessment 2020 (ZIMPHIA 2020): Final Report. Harare, Zimbabwe: MOHCC, 2021.
